# Baseline PTSD and depressive symptoms predict multidimensional recovery after combat-related transtibial amputation: a prospective cohort study

**DOI:** 10.3389/fresc.2026.1891994

**Published:** 2026-09-10

**Authors:** Iryna Halabitska, Iryna Kamyshna, Mykhailo Buchynskyi, Pavlo Petakh, Oleksandr Kamyshnyi

**Affiliations:** 1 Department of Therapy and Family Medicine, I. Horbachevsky Ternopil National Medical University, Ternopil, Ukraine; 2 Department of Medical Rehabilitation, I. Horbachevsky Ternopil National Medical University, Ternopil, Ukraine; 3 Department of Microbiology, Virology, and Immunology, I. Horbachevsky Ternopil National Medical University, Ternopil, Ukraine; 4 Department of Biochemistry and Pharmacology, Uzhhorod National University, Uzhhorod, Ukraine

**Keywords:** functional recovery, post-traumatic stress disorder, postural control, prosthetic gait, psychiatric comorbidity, robotic neurorehabilitation, transtibial amputation

## Abstract

**Introduction:**

Posttraumatic stress disorder (PTSD) is common among combat-injured service members. To date, there is insufficient evidence to determine whether the initial severity of psychiatric disorders is associated with the dynamics of motor recovery after lower limb amputation. The aim of the study was to assess whether the severity of PTSD and depressive symptoms at the time of hospitalization affect the outcomes of intensive robotic neurorehabilitation after combat-related unilateral transtibial amputation.

**Methods:**

A prospective cohort study included 45 male service members with unilateral transtibial amputations who underwent a standardized robotic neurorehabilitation program for the lower limbs. The examinations were performed at four time points: on days 0, 14, 30, and 120. 46 indicators were evaluated, including stabilometric posturography (Stabiloplatforma Alfa), instrumental gait analysis (OMEGO, STRIDE ONE, BALO), clinical functional tests (BBS, TUG, FRT, AMPPRO, FSST, 10MWT, 2MWT, 6MWT, ABC), pain intensity (VAS), as well as psychiatric scales PCL-5 and PHQ-9. Statistical analyses included longitudinal mixed-effects modeling, multivariable regression, and exploratory machine-learning approaches.

**Results:**

The study found that the level of PTSD and depression at the beginning of rehabilitation was associated with functional recovery after combat transtibial amputation. 37.8% of participants recorded a clinically significant improvement according to the combined criterion. Psychiatric scales (PCL-5 and PHQ-9) showed moderate predictive ability for rehabilitation response (AUC = 0.80). The PCL-5, PHQ-9, VAS, and baseline functional indicators (BBS and TUG) had the greatest contribution to prediction. The probability of a positive response to treatment decreased with higher PCL-5 values. Cluster analysis identified two types of patients: with high psychiatric burden and worse recovery and with lower burden and better outcomes.

**Conclusions:**

The results suggest that psychiatric status at the beginning of rehabilitation may be an important factor associated with functional recovery after amputation. Screening for PTSD and depression may help predict rehabilitation outcomes. However, these findings need to be confirmed in larger studies. It is also worthwhile to examine whether early psychiatric intervention can improve rehabilitation outcomes.

## 1 Introduction

Combat amputations of the lower extremities remain one of the main causes of long-term disability among military personnel (1–3). Transtibial amputations are the most common type of severe combat injuries of the lower extremities and are accompanied by significant functional limitations, impaired balance, mobility and endurance (4–8). Recovery from such injuries requires a long process of adaptation of motor control, which is disrupted due to the loss of sensory and motor connections (9–11). In modern rehabilitation practice, robotic lower limb rehabilitation systems are increasingly used, which provide structured gait training, sensorimotor stimulation and the possibility of gradual restoration of functional activity of patients (12–14).

Post-traumatic stress disorder (PTSD) is one of the most common mental consequences of combat trauma and is detected in 30%–80% of patients with amputations (15–17). In addition to physical limitations, patients often face long-term psycho-emotional disorders that can negatively affect the effectiveness of rehabilitation (18–20). There are several possible mechanisms for this effect. Chronic stress can disrupt the processes of neuroplasticity and motor learning, constant hypervigilance can reduce the concentration of attention necessary for safe use of the prosthesis (21–23), and depressive symptoms can reduce motivation for regular and long-term training (24–27). At the same time, the role of mental disorders in shaping the results of physical rehabilitation after combat amputations remains insufficiently studied (28, 29).

To date, most studies in this area have focused mainly on functional recovery or separately on the psychiatric consequences of combat trauma (30, 31). However, the relationship between the initial severity of PTSD, depressive symptoms, and long-term dynamics of motor recovery in patients undergoing robotic neurorehabilitation remains insufficiently characterized (32, 33). There is also limited evidence on the use of psychiatric screening to predict rehabilitation outcomes.

Previous studies have demonstrated that psychological factors play an important role in adaptation to limb loss and rehabilitation outcomes. PTSD has been associated with reduced mobility, increased pain perception, lower quality of life, and poorer participation in rehabilitation programs (34–36). Depressive symptoms may negatively influence motivation, adherence to rehabilitation, and prosthetic adaptation (37, 38). Nevertheless, prospective studies evaluating the influence of psychiatric burden on objective functional recovery after combat-related amputation remain limited.

To address this issue, we conducted a prospective observational study with four time points and multimodal analysis of the results. We investigated two main questions. First, is the baseline level of PTSD on the PCL-5 scale associated with the nature and severity of changes in balance, mobility, endurance, posturographic indicators, and gait parameters during a 120-day robotic neurorehabilitation program? Second, is it possible to identify clinically meaningful phenotypes of rehabilitation recovery based on psychiatric screening at hospitalization. Because the study was observational and did not include a control group, the results obtained should be interpreted as associative rather than causal.

## 2 Materials and methods

### 2.1 Study design and participants

A prospective longitudinal observational cohort study was conducted in 45 male military personnel with unilateral transtibial amputations resulting from combat injuries. All participants underwent a standardized robotic neurorehabilitation program of the lower extremities. Patient assessments were performed at four pre-specified time points: day 0 (baseline), day 14, day 30, and day 120 (endpoint). Inclusion criteria were as follows: unilateral transtibial amputation resulting from explosive or gunshot combat injuries, completed primary prosthetics, medical clearance for intensive rehabilitation, and no prior experience in robotic rehabilitation programs. All patients provided written informed consent to participate in the study. The study was approved by the Bioethics Commission of the I. Horbachevsky Ternopil National Medical University (protocol No. 81 dated April 3, 2025) and conducted in accordance with the principles of the Declaration of Helsinki.

No participants were lost to follow-up during the study period. Complete outcome data were available at all assessment time points; therefore, no missing-data imputation procedures were required. Due to the exploratory nature of the study and the absence of reliable preliminary effect-size estimates for this population, an *a priori* sample size calculation was not performed.

### 2.2 Baseline cohort characteristics

All participants were male, with a median age of 42.3 years [32.4–53.1]. In the group with a PCL-5 PTSD score <33, the median age was 41.8 years [31.7–52.0], while in the group with a PCL-5 score ≥33, it was 43.1 years [34.2–54.4] (*p* = 0.68).

The median time since amputation in the overall cohort was 6.2 months [4.1–9.3], including 5.9 months [4.0–8.2] in the PCL-5 score <33 and 6.8 months [4.3–10.1] in the PCL-5 score ≥33 (*p* = 0.74).

Left lower limb amputation occurred in 46.7% of participants overall (45.0% in the PCL-5 < 33 group and 48.0% in the PCL-5 ≥ 33 group), and right lower limb amputation occurred in 53.3% (55.0% and 55.0%, respectively; *p* = 0.81). All patients were already using a prosthesis at the time of inclusion.

Analgesic therapy was comparable between groups, and antidepressants were not used by any participant during the follow-up period.

All participants used contemporary energy-storing prosthetic feet and modular prosthetic systems. Socket types included predominantly total surface bearing and patellar tendon bearing designs according to individual clinical requirements. Residual limb condition was clinically stable in all participants at enrollment, with no active wound complications. Detailed prosthetic component-level analysis was unavailable and should be considered in future studies.

### 2.3 Robotic neurorehabilitation protocol

In accordance with contemporary evidence-based rehabilitation standards, including the Ministry of Health of Ukraine Standard No. 1868 (2025) (39), the VA/DoD Clinical Practice Guideline for the Rehabilitation of Lower and Upper Limb Amputation (2022) (40), and the BACPAR Toolbox v2 (2014) (41), the rehabilitation protocol was developed following a multidisciplinary, task-oriented framework for lower limb amputation rehabilitation and included structured robotic gait training combined with traditional physical therapy. Each session lasted 45–60 min and was conducted five times a week under the direct supervision of certified rehabilitation professionals experienced in prosthetic gait training. The intensity of the training and the difficulty of the tasks were gradually modified individually depending on the patient's load tolerance, balance, gait stability, pain level, and functional outcomes of previous sessions. The criteria for progression were improved walking endurance, decreased gait asymmetry, improved postural control, and the ability to safely perform more complex locomotor tasks. Compliance with the rehabilitation program was monitored using visit logs and records completed by therapists; all participants completed more than 90% of the scheduled sessions. Robotic rehabilitation was performed using robotic lower limb gait training systems for patients with lower limb amputations and traumatic lower limb injuries, including assessment and training on the OMEGO®, StrideOne, and THERA-Trainer Balo platforms, as well as the Alfa stabilization platform. These systems were integrated with body weight support systems and real-time feedback technologies, which facilitated motor function retraining, improved balance, and safe prosthetic ambulation.

The rehabilitation program consisted of progressive balance training, weight-shifting exercises, gait symmetry correction, obstacle negotiation, treadmill-assisted walking, dynamic postural control exercises, and task-specific prosthetic gait training. Progression criteria included achievement of predefined stability and endurance targets. Therapists continuously adjusted task complexity and body-weight support according to individual performance.

### 2.4 Outcome assessments

#### 2.4.1 Stabilometric posturography (Stabiloplatforma Alfa)

Static postural control was assessed using three test paradigms. The Romberg test was used to record the dynamics of the center of pressure (COP) during bipedal standing with eyes open (EO) and closed (EC). Twelve parameters were assessed: COP displacement along the *X* and *Y* axes (cm), mean COP velocity in mediolateral and anteroposterior directions (cm/s), area of oscillation (cm^2^), and trajectory length (cm), which were determined separately in the EO and EC conditions. The Unterberger test was used to assess vestibular-associated angular deviation per step (°) and movement amplitude (°). The movement time test was used to determine the speed of sensorimotor information processing along the four diagonals (ms).

#### 2.4.2 Technology-assisted gait analysis

Three systems provided a comprehensive assessment of functional parameters. The OMEGO system was used to determine weight distribution (%), proprioceptive difference (°), and limb loading force (kg). The STRIDE ONE system recorded the smoothness index, symmetry index (%), foot roll quality (%), stance phase ratio (%), and stride duration (s). The BALO system assessed anteroposterior, mediolateral, and lateral stability. All parameters were determined at days 0, 14, 30, and 120 of the study.

#### 2.4.3 Clinical functional tests

Standardized clinical assessments included the Berg Balance Scale (BBS), the Timed Up and Go (TUG), the Functional Reach Test (FRT), the Amputee Mobility Prediction Scale (AMPPRO), the Four-Square Test (FSST), the 10-Meter Walk Test (10MWT), the 2-Minute Walk Test (2MWT), the 6-Minute Walk Test (6MWT), the Activity-Based Balance Confidence Scale (ABC), and the Visual Analogue Pain Scale (VAS). All tests were administered at each stage of the study.

#### 2.4.4 Psychiatric assessments

PTSD symptoms were assessed using the PCL-5 (range 0–80; ≥33 indicating probable PTSD). Depressive symptoms were quantified using the PHQ-9 (range 0–27; ≥15 indicating moderately severe-severe depression). Baseline pain was stratified by VAS (0–3 mild; 4–6 moderate). Psychiatric instruments were administered at Days 0 and 120.

### 2.5 Statistical analysis

The normality of the data distribution was assessed using the Shapiro–Wilk test. Since most of the measures did not follow a normal distribution, nonparametric methods were used for the main analysis. Data are presented as median and interquartile range [Q1–Q3]. Changes in measures within groups were assessed using the Wilcoxon paired-rank test, and overall longitudinal changes were assessed using the Friedman test. The Mann–Whitney *U* test was used to compare groups at each time point. Effect sizes are reported as Cohen's *d* for the distribution of changes in measures. Although non-parametric tests were used for most analyses, Cohen's *d* was additionally reported to facilitate comparison with previous rehabilitation studies where standardized mean differences remain commonly used.

Stratified analyses were performed using three binary classifiers: PCL-5 level (≥33 vs. <33), PHQ-9 level (≥15 vs. <15), and VAS level (4–6 vs. 0–3). For each stratified analysis, false discovery rate (FDR) correction was applied using the Benjamini-Hochberg method; results reported in the main text corresponded to a *q* < 0.05 level.

To assess the independent effect of baseline PCL-5 on rehabilitation outcomes, multivariable linear regression models were constructed for the three main functional measures of change (ΔBBS, ΔTUG, Δ6MWT) while controlling for demographic and clinical factors. Each model included baseline PCL-5 score (continuous), age, time since amputation, side of amputation, baseline VAS score, and the corresponding baseline functional measure. Variance inflation factor (VIF) was estimated for all predictors. Due to the high correlation between PCL-5 and PHQ-9 (Spearman's *ρ* = 0.73), a separate set of models was constructed in which PHQ-9 replaced PCL-5 to avoid collinearity. The results are presented as standardized *β*-coefficients with 95% confidence intervals. The residuals of the models were additionally checked for heteroscedasticity and the presence of influential observations.

To assess the differences in the dynamics of indicators between psychiatric subgroups, Generalized Linear Mixed Models (GLMM) were utilized for each of the 46 variables to account for the non-normal distribution of the continuous data. The models were constructed using a Gamma probability distribution with an identity link function. Time (0, 14, 30, and 120 days) and psychiatric subgroup (PCL-5 and PHQ-9 levels) were included as fixed effects, while patient identifier was specified as a random intercept to account for repeated measures. Two models were built in parallel for each variable using PCL-5 and PHQ-9 levels as between-group factors. The main indicator of the analysis was the interaction “time × group”. The level of statistical significance was set at *p* < 0.05.

Several additional exploratory analyses were performed, the results of which are presented for hypothesis-building purposes only and are presented in the Supplementary Tables S6 and S7. These included ROC analysis to predict the composite measure of rehabilitation efficacy (BBS ≥5 points and TUG reduction ≥5 s at day 120), Random Forest regression to assess the contribution of psychiatric predictors to rehabilitation outcomes, *k*-means clustering for baseline variables, LOESS-smoothed treatment response curves, bootstrap mediation analysis, and Elastic Net regression. These analyses are exploratory in nature and did not adjust for multiple comparisons, so the results should be interpreted with caution given the small sample size.

Given the relatively small sample size, the primary confirmatory analyses were predefined and included longitudinal within-group comparisons, stratified analyses, multivariable regression models, and GLMM. Machine-learning approaches (Random Forest, Elastic Net), clustering, ROC analyses, and mediation analyses were conducted exclusively as exploratory hypothesis-generating procedures. These exploratory analyses should not be interpreted as definitive predictive models and require external validation in larger independent cohorts.

Retrospective power analyses based on the effect sizes obtained showed that the sample of 45 participants provided high power (>97%) for the main within-group effects of rehabilitation (Cohen's *d* = 0.59–1.86). For between-group comparisons stratified by PCL-5 level (*n* = 20 vs. 25), the power was 91.8%–99.2% for the effect sizes found. However, it should be noted that the absolute sample size was small, so the study had limited power to analyze interactions, mediation models, and high-dimensional predictor selection. Detailed power calculations are provided in Supplementary Table S5. Additional LOESS dose-response curves and retrospective power analyses are shown in Supplementary Figure S2. All analyses were performed in JASP (v0.18.3), GraphPad Prism (v8.1), and Python 3.12 (scipy, scikit-learn, statsmodels).

## 3 Results

### 3.1 Baseline cohort characteristics

**Figure 1:**
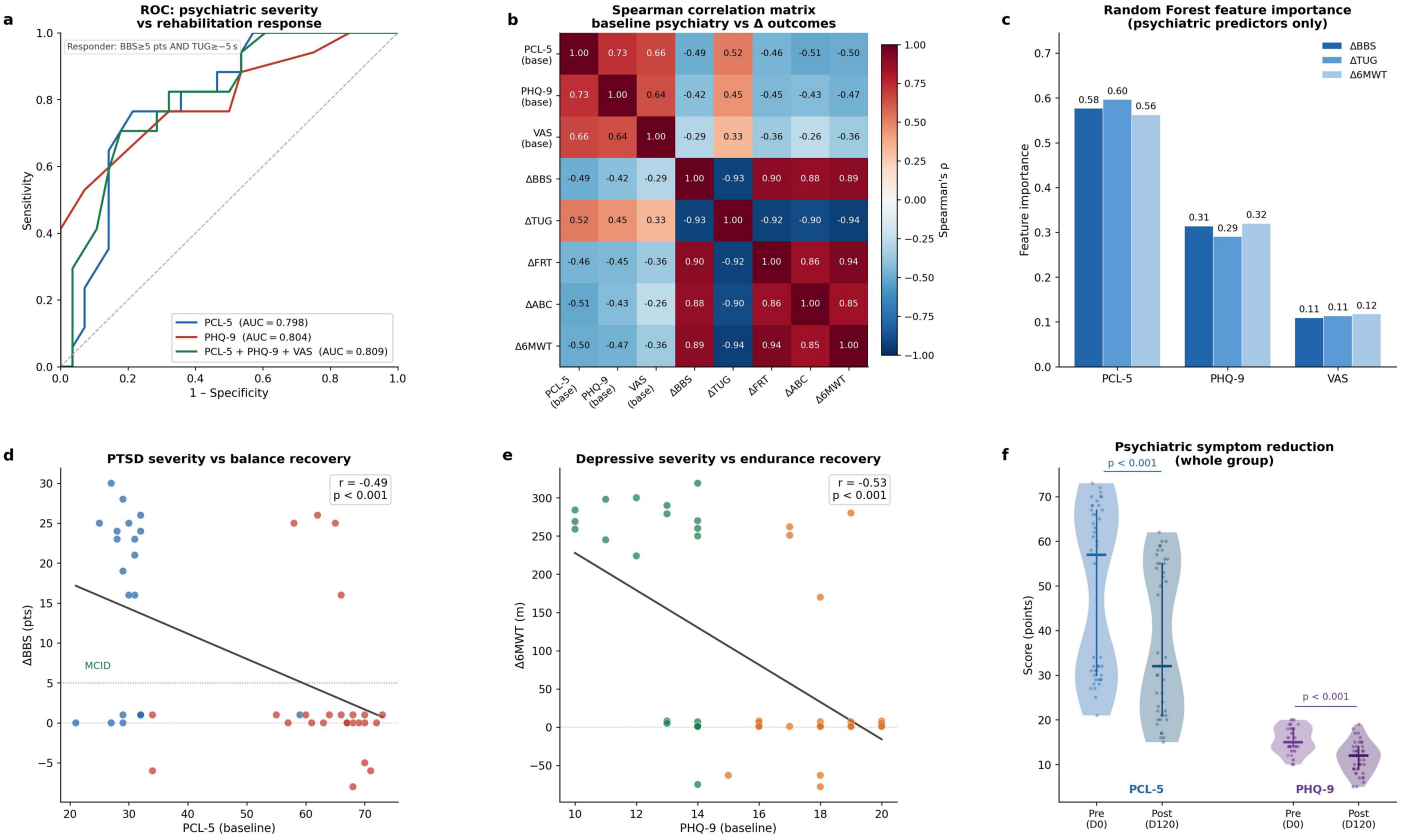
Correlations between baseline psychiatric severity and rehabilitation outcomes. **(a)** ROC curves shown for visualization purposes (exploratory analysis; see Supplementary Table S6). **(b)** Spearman correlation matrix between baseline psychiatric predictors and change scores; PCL-5-PHQ-9 correlation: *ρ* = 0.73. **(c)** Random Forest feature-importance estimates (exploratory; see Supplementary Table S6). **(d)** Scatter plot of baseline PCL-5 against ΔBBS, with regression line and MCID reference (dashed line = 5 pts). **(e)** Scatter plot of baseline PHQ-9 against Δ6MWT, with regression line. **(f)** Pre-post violin plots for PCL-5 and PHQ-9 (whole group); Wilcoxon *p* < 0.001 for both..

The study included 45 male servicemen with unilateral transtibial amputation due to combat trauma. The median PCL-5 score was 57.0 [30.0–67.0] points; 25 participants (55.6%) met the criteria for probable PTSD (≥33 points). The median PHQ-9 score was 15.0 [14.0–18.0] points, with 23 participants (51.1%) scoring ≥15 points. The median VAS score was 4.0 [3.0–5.0] points. There was a strong correlation between the PCL-5 and PHQ-9 scores (Spearman's *ρ* = 0.73; *p* < 0.001; Figure 1B). Demographics (age, time since amputation, and side of amputation) were not statistically different between groups stratified by PCL-5 level.

### 3.2 Whole-group rehabilitation outcomes

**Figure 2:**
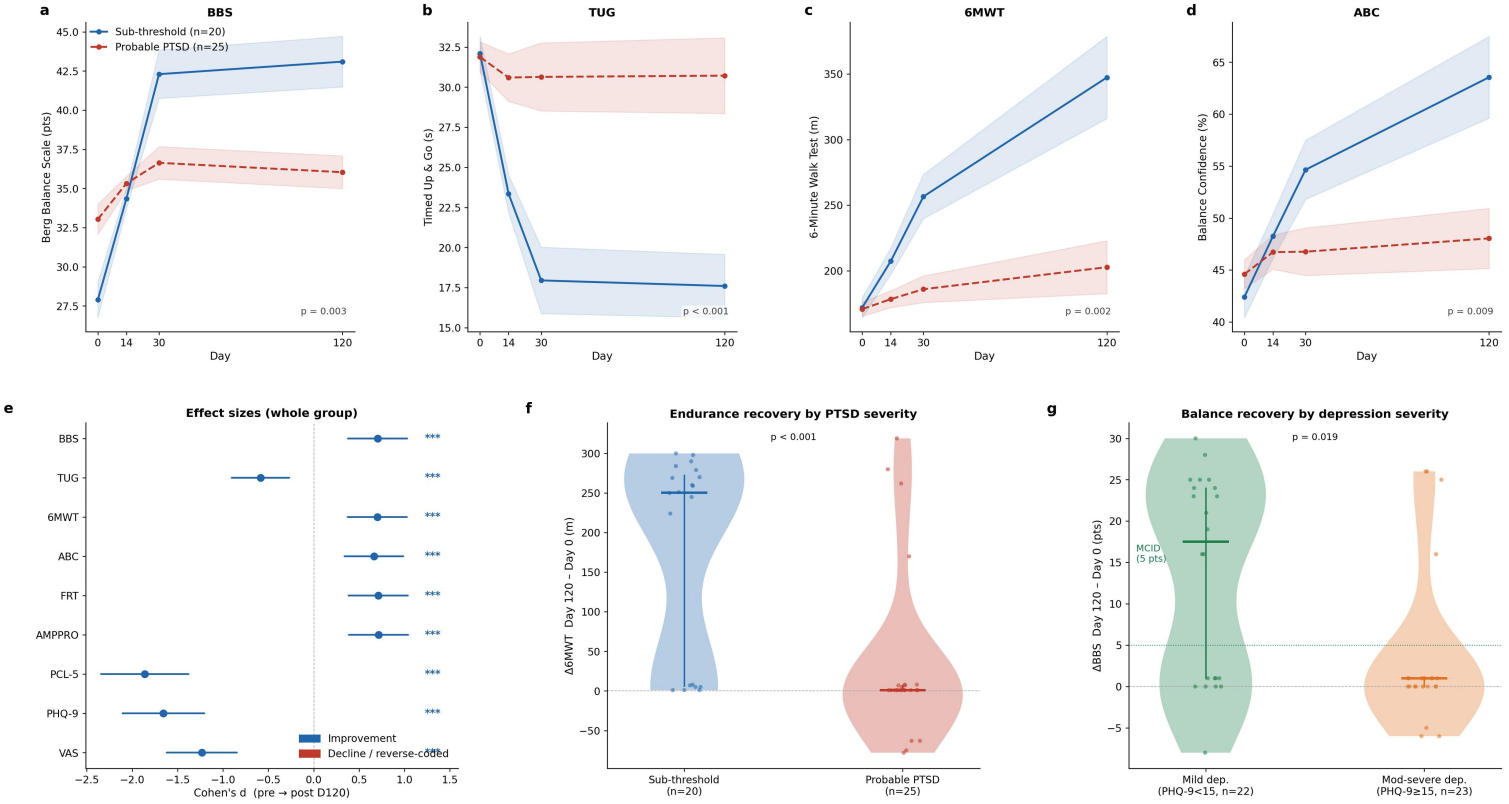
Rehabilitation trajectories, effect sizes, and outcome divergence. **(a–d)** Mean (± SEM) longitudinal trajectories for BBS, TUG, 6MWT, and ABC across Days 0, 14, 30, and 120, stratified by baseline PCL-5 level. Blue solid line: PCL-5 < 33 (*n* = 20). Red dashed line: PCL-5 ≥ 33 (*n* = 25). Shaded regions: ± 1 SEM. Mann–Whitney *U* at Day 120: all *p* < 0.005. **(e)** Forest plot of Cohen's *d* (95% CI) for primary outcomes (whole group, Day 0 vs. Day 120). Asterisks: *p < 0.05; **p < 0.01; ***p < 0.001 (Wilcoxon signed-rank). **(f)** Violin and jitter plot of Δ6MWT by PCL-5 level. **(g)** Violin and jitter plot of ΔBBS by PHQ-9 level; dashed green line = MCID (5 points). Complete data are reported in Supplementary Table S1.

In the entire cohort, the 120-day robotic neurorehabilitation program was associated with statistically significant improvements in all 46 measures studied (Supplementary Table S1). The median PCL-5 decreased from 57.0 to 32.0 points (Cohen's *d* = −1.86; *p* < 0.001), PHQ-9 from 15.0 to 12.0 points (*d* = −1.66; *p* < 0.001), and VAS from 4.0 to 2.0 points (*d* = −1.23; *p* < 0.001; Figure 1F). Improvements in functional measures were also noted: median changes in BBS were +1.0 points (*d* = 0.70), TUG −1.0 s (*d* = −0.59), FRT +1.0 cm (*d* = 0.71), 6MWT +7.0 m (*d* = 0.70), and AMPPRO +2.0 points (*d* = 0.71; all *p* < 0.001). Forest plots of all effect sizes are shown in Figure 2E. Friedman's test confirmed statistically significant changes between the four time points for all main measures (all *p* < 0.001).

### 3.3 Posturographic and gait changes

**Figure 3:**
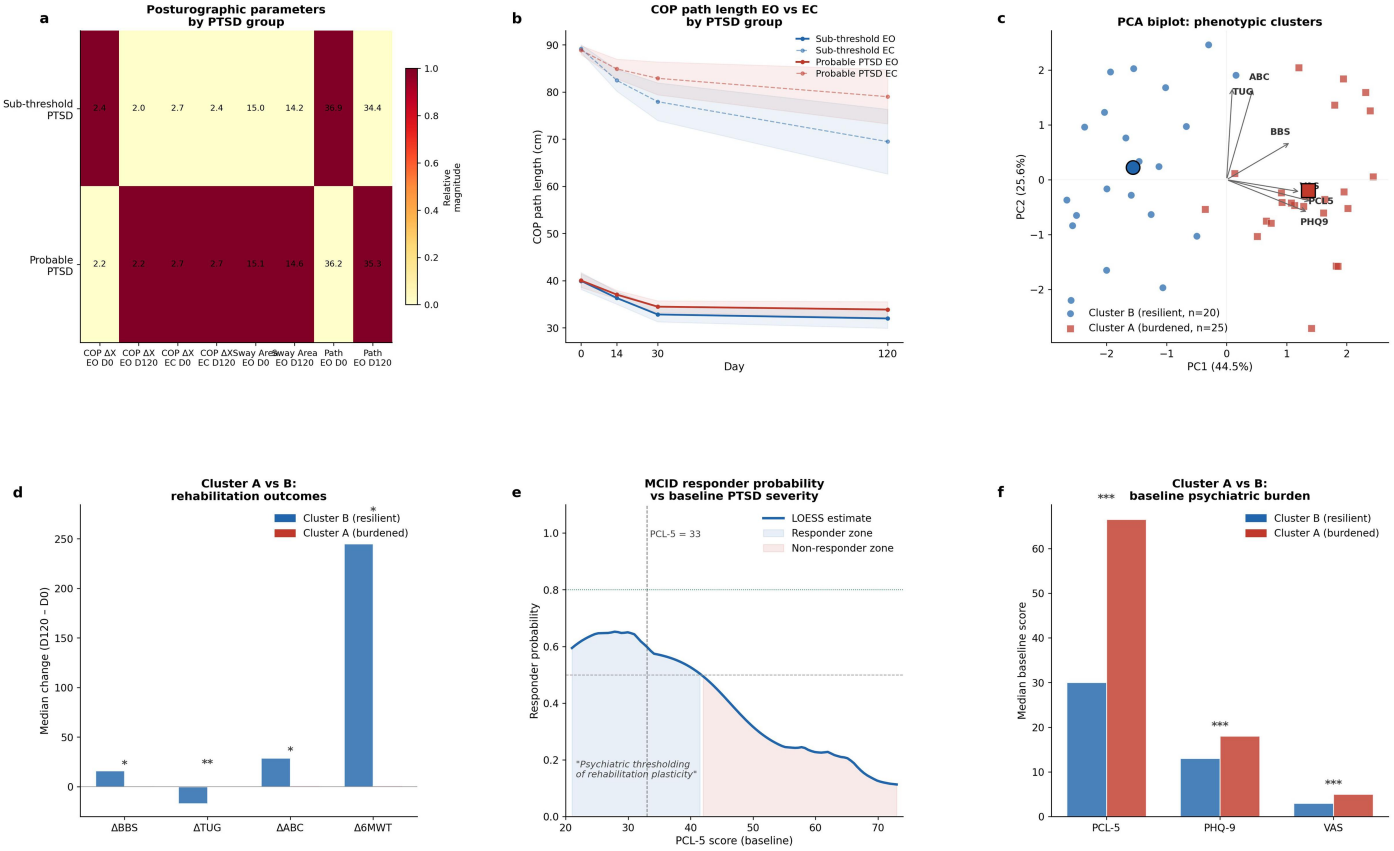
Posturographic and exploratory cluster analyses. **(a)** Heat map of posturographic median parameters (COP displacement, velocity, area of oscillation, path length; EO and EC conditions) by PCL-5 group. **(b)** COP path length trajectories (EO and EC) by PCL-5 group across Days 0–120. **(c)** PCA biplot of *k*-means clusters (*k* = 2) with 95% confidence ellipses; Cluster A: higher psychiatric burden (*n* = 25); Cluster B: lower psychiatric burden (*n* = 20). **(d)** Median rehabilitation change scores (ΔBBS, ΔTUG, ΔABC, Δ6MWT) by cluster; asterisks indicate between-cluster significance (Mann–Whitney *U*). **(e)** LOESS-smoothed probability of MCID response (BBS ≥ 5 pts and TUG reductio*n* ≥ 5 s) as a function of baseline PCL-5 score; dashed vertical line = PCL-5 threshold of 33. **(f)** Median baseline psychiatric scores (PCL-5, PHQ-9, VAS) by cluster. All cluster analyses are exploratory (see Supplementary Table S7) and results are not adjusted for multiple comparisons.

Stabilometric analysis demonstrated a small but statistically significant reduction in static postural oscillations (Supplementary Tables S1, S2; Figure S3). Under eyes-open conditions, the COP *X*-axis displacement decreased from 2.24 to 2.18 cm, the oscillation area from 15.10 to 14.50 cm^2^, and the COP trajectory length from 89.5 to 87.3 cm (all *p* < 0.001). The STRIDE ONE symmetry index improved from 59.4% to 85.1% (*p* < 0.001), while the proprioceptive difference decreased from 71.0° to 36.0° (*p* < 0.001). Posturographic trajectories by PCL-5 group are shown in Figures 3A,B.

### 3.4 Baseline PCL-5 level was associated with smaller functional gains

Stratification by baseline PCL-5 level showed significantly smaller gains in functional scores in the probable PTSD group compared with the subthreshold PTSD group across all functional domains (Figures 2A–D,F; Supplementary Table S2). Participants with subthreshold PTSD (*n* = 20) demonstrated a median ΔBBS of 20.0 points vs. little or minimal improvement in the probable PTSD group (*n* = 25; *p* = 0.001), ΔTUG of −17.5 s vs. minimal change (*p* < 0.001), ΔFRT of 10.0 cm vs. minimal change (*p* < 0.001), ΔAMPPRO of 20.5 points vs. little improvement (*p* = 0.001), ΔABC of 29.5% vs. minimal gain (*p* < 0.001), and Δ6MWT of 250.5 m vs. little gain (*p* < 0.001). The dynamics of the measures across all four time points are shown in Figures 2A–D, while individual spaghetti plots according to PCL-5 quartiles are presented in Supplementary Figure S1.

### 3.5 Multivariable adjusted analyses

In multivariable linear regression models adjusted for age, time since amputation, side of amputation, baseline VAS score, and baseline functional measures, baseline PCL-5 remained independently associated with changes in ΔBBS (standardized *β* = −0.49; 95% CI: −0.71 to −0.27; *p* < 0.001), ΔTUG (*β* = 0.42; 95% CI: 0.18–0.66; *p* = 0.001), and Δ6MWT (*β* = −0.55; 95% CI: −0.78 to −0.32; *p* < 0.001). In parallel models substituting the PCL-5 for the PHQ-9, baseline PHQ-9 was also independently associated with all three measures of change (all *p* < 0.01), with similar effect sizes. VIF values were below 2.5 for all predictors in both sets of models, and analysis of residuals revealed no significant heteroscedasticity. The results are presented in Table 1. Scatter plots illustrating the relationship between baseline PCL-5 and PHQ-9 scores and changes in BBS and 6MWT are shown in Figures 1D,E; ROC curves and Random Forest importance estimates are presented for visualization purposes in Figures 1A,C.

**Table 1:** Multivariable linear regression: association of baseline psychiatric severity with rehabilitation change scores.

Outcome	Predictor	*β* (95% CI)	*p*	VIF
ΔBBS	PCL-5 (baseline)	−0.49 (−0.71, −0.27)	<0.001	2.1
	PHQ-9 (baseline)	−0.45 (−0.68, −0.22)	<0.001	2.0
ΔTUG	PCL-5 (baseline)	0.42 (0.18, 0.66)	0.001	2.2
	PHQ-9 (baseline)	0.38 (0.14, 0.62)	0.003	2.1
Δ6MWT	PCL-5 (baseline)	−0.55 (−0.78, −0.32)	<0.001	2.0
	PHQ-9 (baseline)	−0.51 (−0.74, −0.28)	<0.001	1.9

Standardized *β* coefficients with 95% confidence intervals. Models adjusted for age, time since amputation, side of amputation, baseline VAS, and the corresponding baseline functional score. PCL-5 and PHQ-9 fitted in separate models due to collinearity (*ρ* = 0.73).

### 3.6 Depressive severity and pain

Stratification by PHQ-9 score (≥15 vs. <15) showed smaller gains in BBS, TUG, 6MWT, and ABC scores in the moderate and severe depression group (all between-group *p* < 0.01 at day 120; Supplementary Table S3). Changes in ΔBBS by PHQ-9 score are shown in Figure 2G. Stratification by VAS score (moderate vs. mild pain) also showed similar between-group differences (Supplementary Table S4).

### 3.7 Trajectory-level analysis: GLMM

**Figure 4:**
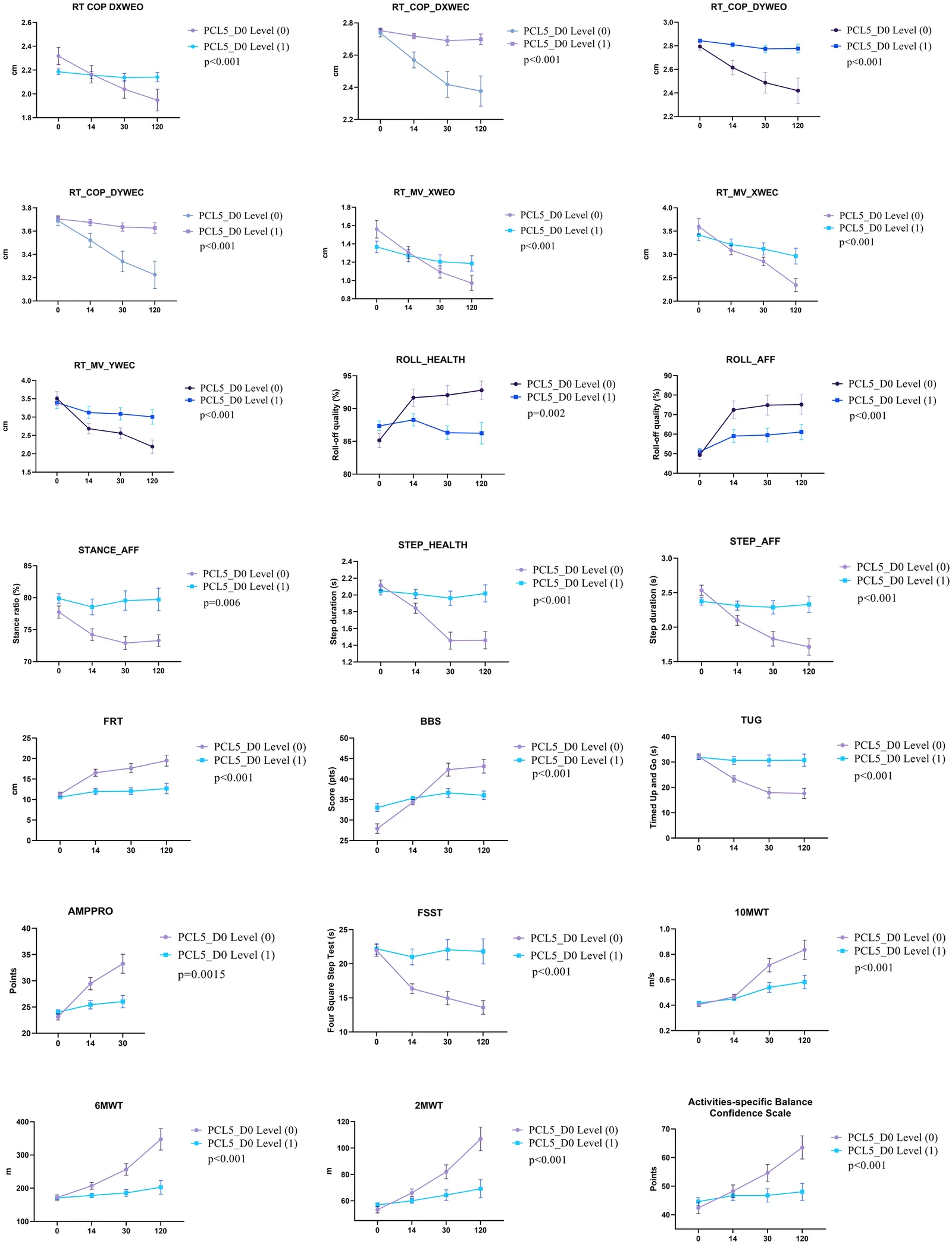
Time × psychiatric level interactions across rehabilitation outcomes (GLMM): PCL-5 stratification. PCL-5 stratification across Romberg Test stabilometric parameters, gait parameters and clinical functional tests. All sub-panels show mean ± SEM at Days 0, 14, 30, and 120. *P* value indicates the Time × Group interaction; The models were constructed using a Gamma probability distribution with an identity link function. These results describe associations between baseline PTSD severity and longitudinal change patterns and do not establish causal effects.

**Figure 5:**
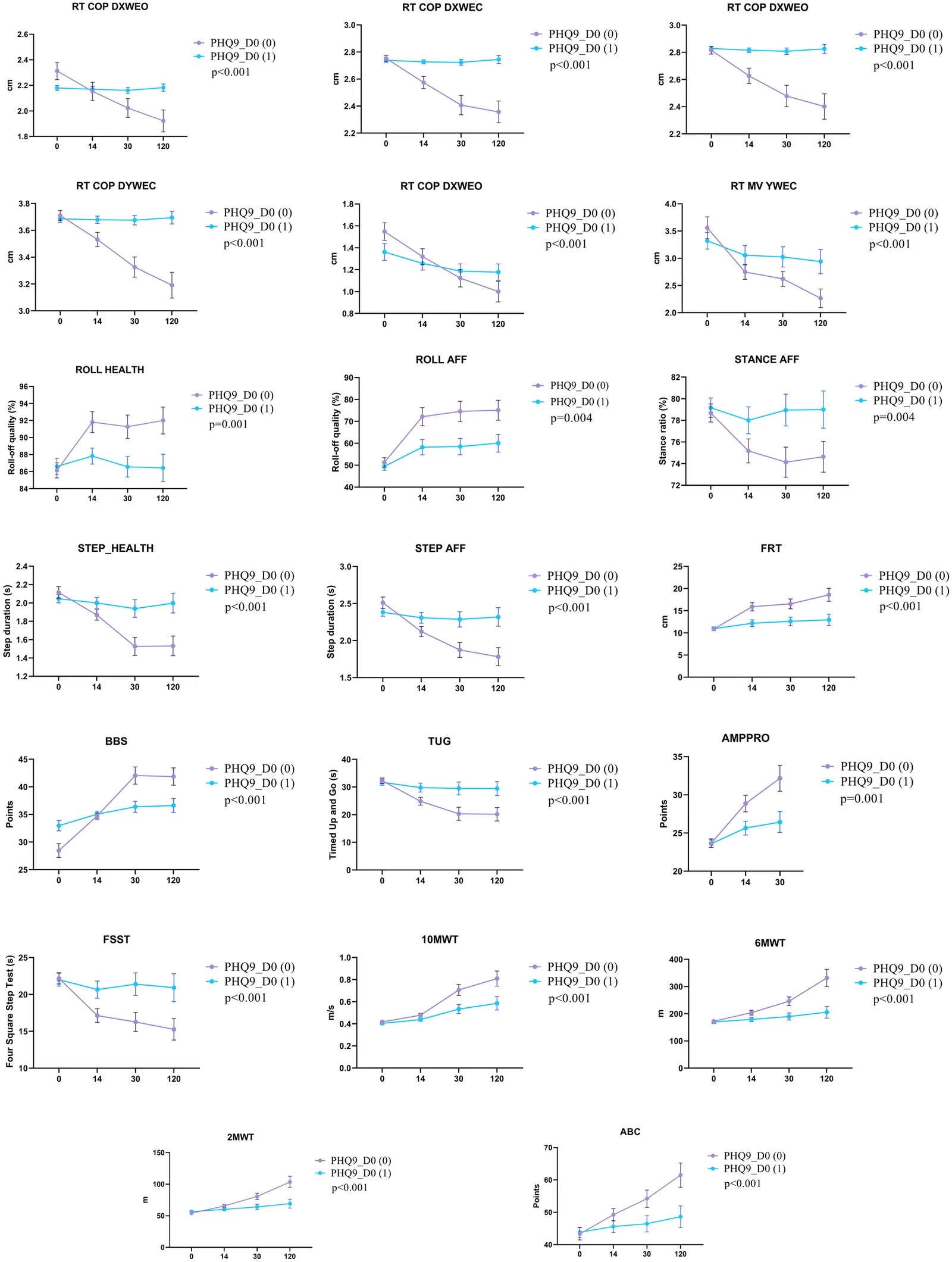
Time × psychiatric level interactions across rehabilitation outcomes (GLMM): PHQ-9 stratification. **(a)** PHQ-9 stratification across Romberg Test stabilometric parameters, gait parameters and clinical functional tests. All sub-panels show mean ± SEM at Days 0, 14, 30, and 120. *P* value indicates the Time × Group interaction; the models were constructed using a Gamma probability distribution with an identity link function. These results describe associations between baseline depressive symptom burden and longitudinal change patterns and do not establish causal effects.

Two-Way Repeated Measures ANOVA was used to test whether the two psychiatric strata had different trajectories of change over time (Figures 4,5). The interaction of “time × PCL-5 level” was statistically significant for most posturographic, gait, and clinical-functional measures. In particular, for the lateral displacement of COP in the eyes-open condition (*p* < 0.001; Figure 4), in the eyes-closed condition (*p* < 0.001), the average velocity of COP along the *X* axis (*p* < 0.001), the quality of the roll on the healthy limb (*p* = 0.002) and on the affected limb (*p* < 0.001), as well as the duration of the step on both limbs (both *p* < 0.001).

Within the clinical functional tests, significant interactions were noted for BBS, TUG, FSST, 6MWT, 2MWT, ABC, FRT, 10MWT (all *p* < 0.0001) and AMPPRO (*p* = 0.0015).

Stratification by PHQ-9 level showed a similar pattern (Figure 5), with significant time × PHQ-9 level interactions for COP lateral deviation (eyes open and eyes closed: *p* < 0.001), as well as for the main clinical functional tests (BBS, TUG, FSST, 6MWT, ABC, FRT, 10MWT; all *p* < 0.001). Taken together, the results of the GLMM indicate that baseline psychiatric burden is associated with different trajectories of change over time in most of the indicators studied. However, these results describe associations rather than proving causality.

### 3.8 Exploratory predictive analyses

Among the 45 participants, 17 (37.8%) met the combined treatment efficacy criterion (improvement in BBS score ≥5 points and reduction in TUG time ≥5 s). ROC analysis demonstrated moderate discriminatory power of the models: AUC was 0.798 for PCL-5, 0.804 for PHQ-9 and 0.809 for the combined psychiatric index, suggesting the potential suitability of these measures for identifying patients who would respond to rehabilitation at the preliminary analysis stage (Figure 1A, Supplementary Table S6). Random forest analysis showed that the baseline PCL-5 score had the highest normalized feature importance for predicting rehabilitation outcomes (56.9% for ΔBBS, 59.0% for ΔTUG and 56.0% for Δ6MWT), followed by PHQ-9 (29.9%–32.7%) and VAS (10.7%–11.3%; Figure 1C, Supplementary Table S6). The relationship between baseline psychiatric scores and motor function changes is shown in Figures 1D,E.

The probability of a positive response to treatment exceeded 80% for PCL-5 scores below 30 and decreased to less than 20% for scores above 55. The greatest reduction was observed in the range of 30–45 points (Figure 3E; Supplementary Figure S2a). The results indicate that higher levels of PTSD symptoms at baseline were associated with a lower probability of achieving clinically meaningful functional improvement according to the predefined response criterion.

The k-means clustering method (*k* = 2) allowed us to distinguish two clinically distinct rehabilitation profiles (Figures 3C,D,F; Supplementary Table S7). Cluster A (*n* = 25), which was characterized by higher psychiatric burden, had mean PCL-5 = 62.8 ± 10.2, PHQ-9 = 17.4 ± 2.0, and VAS = 5.0 ± 0.9, and limited functional improvement (median ΔBBS = 4.0 points; Δ6MWT = 42.0 m). Cluster B (*n* = 20), which was characterized by lower psychiatric burden, had mean PCL-5 = 31.1 ± 8.7, PHQ-9 = 13.0 ± 1.9, and VAS = 2.4 ± 0.8, and significantly better rehabilitation outcomes (median ΔBBS = 14.0 points; Δ6MWT = 162.9 m). The difference between clusters in ΔBBS was statistically significant (Mann–Whitney test, *p* = 0.006). Principal component analysis to assess cluster separation is shown in Figure 3C, and cluster-level changes in rehabilitation measures are shown in Figure 3D.

In the elastic network regression cross-validation, the PCL-5, PHQ-9, VAS, baseline BBS, and baseline TUG were included in the model in more than 80% of the 100 bootstrap iterations. However, posturographic and instrumental gait parameters were included infrequently and inconsistently (selection rate <30%) (Supplementary Table S8). This set of five measures, which includes two psychiatric scales, one pain measure, and two baseline functional tests, may serve as a preliminary basis for further prospective validation.

## 4 Discussion

In this prospective observational study of 45 combat-related transtibial amputees who underwent standardized robotic neurorehabilitation for 120 days, baseline PTSD severity was independently associated with smaller functional improvements in all major domains, including balance, mobility, endurance, posturographic, and gait parameters. This association persisted after adjustment for age, time since amputation, side of lesion, baseline pain, and baseline functional measures. GLMM analysis further demonstrated that baseline psychiatric burden was associated not only with differences in outcome but also with significantly different recovery trajectories over time, which may indicate a modifying role of mental state in the rehabilitation process.

Importantly, interpretation of statistical significance should be accompanied by consideration of clinical relevance. Improvements observed in BBS, TUG, AMPPRO, and walking-distance measures approached or exceeded published thresholds for clinically meaningful functional improvement in lower-limb amputee populations.

The possible mechanisms for these associations are diverse. Chronic activation of stress systems in PTSD may affect the functioning of the nervous system through changes in neurotrophic signaling and thus slow down motor learning and adaptation to the prosthesis (42–44). Increased alertness and focus on threat may reduce the cognitive resources required for the complex coordination of movements and processing of sensory information during walking with a prosthesis (45–47). Depressive symptoms may further reduce motivation, endurance, and regularity of participation in repetitive training, which is critical for the formation of motor skills (48–50). Increased between-group differences in posturographic indicators under closed eyes may indirectly indicate changes in sensory integration and a decrease in the ability to compensatory use of non-visual balance control strategies (51–53), but these interpretations remain hypothetical and cannot be unequivocally confirmed based on the available data.

The results are consistent with previous studies in patients with stroke, traumatic brain injury, and chronic pain, where psychiatric comorbidity has been associated with slower functional recovery (54–57). However, this study extends the existing knowledge by focusing on a specific cohort of military personnel with combat transtibial amputations and using multivariable outcome assessment with repeated measurements at four time points in combination with robotic neurorehabilitation.

Additional biological and neurophysiological mechanisms may partially explain the observed influence of mental state on functional recovery trajectories after amputation (34, 58, 59). Chronic activation of stress-reactive systems, characteristic of posttraumatic stress disorder, is associated with dysregulation of the hypothalamic-pituitary-adrenal axis, changes in neurotrophic signaling, and impaired neuroplasticity processes that are critical for motor learning and adaptation to prosthetics (60–64). Increased sympathetic activity and a state of hyperarousal may also alter sensorimotor integration, affecting the accuracy of processing proprioceptive and vestibular information during the formation of a new gait pattern during robotic rehabilitation (65–68).

Depressive symptoms can additionally reduce the level of motivation and the ability to engage in long-term repetitive training, which is key to consolidating motor skills (69–72). As a result, this can lead to slower improvement in balance, gait, and posturographic indicators, especially in conditions of complex tasks, such as in the absence of visual control (73–75). Thus, the psychoemotional state of patients can significantly affect the speed and quality of recovery from amputation (35, 76, 77). The presented studies show that the development of systemic diseases depends on a combination of different factors, including metabolic, genetic and immuno-inflammatory processes (78–83). Changes in the functioning of genes and signaling pathways are associated with how the disease progresses in different organs and systems (84–87), including the endocrine, cardiovascular and immune (88–93). Systemic diseases, such as diabetes mellitus, cardiovascular pathologies, and inflammatory lesions of the musculoskeletal system, often have a multifactorial origin and a complex course (94–97). In the context of amputation and subsequent rehabilitation, similar mechanisms may also influence the recovery of patients, in particular through their influence on the plasticity of the nervous system (98–101), the response to stress and the coordination of movements, which are important for learning to walk and adapting to a prosthesis (102–105).

The findings highlight the value of routine psychiatric screening at the time of entry into a rehabilitation program. The use of short validated instruments, such as the PCL-5 and PHQ-9, is feasible in clinical settings and may facilitate early stratification of patients at risk of poorer functional recovery. However, these instruments should not be used for individual prediction without further validation in larger independent samples. A combination of trauma-focused psychotherapy, pharmacological treatment, and intensive robotic rehabilitation may be potentially promising, which may reduce the observed differences between subgroups, but this needs to be verified in randomized trials.

A number of limitations should be considered. The relatively small sample size limits the precision of subgroup estimates, the stability of regression models, and the robustness of complex interaction analyses. The observational design and lack of a control group preclude causal inferences regarding the effects of the rehabilitation program itself and do not allow complete separation of treatment-related improvements from natural recovery. In addition, the study did not include a comparison group of combat-exposed military personnel without major physical injury; therefore, it was not possible to determine whether the prevalence and severity of PTSD symptoms observed in this cohort differed from those associated with combat exposure alone.

The cohort consisted exclusively of male military personnel with unilateral transtibial amputations treated under wartime conditions. Furthermore, all participants underwent an intensive multidisciplinary robotic rehabilitation program requiring specialized equipment and substantial clinical resources. Consequently, extrapolation of these findings to civilian populations, women, other amputation levels, different healthcare systems, and rehabilitation settings with lower resource availability should be performed with caution.

Psychiatric assessments (PCL-5 and PHQ-9) were conducted only at baseline and day 120. As a result, the intermediate trajectories of PTSD and depressive symptoms during rehabilitation could not be characterized, and potential fluctuations in psychological status over time may have been missed. The high correlation between psychiatric scales also limits the ability to disentangle the independent contributions of PTSD and depressive symptoms.

Although posturographic and instrumental gait variables were comprehensively collected, these parameters demonstrated relatively low selection stability in the exploratory predictive models and were included inconsistently compared with psychiatric and clinical functional measures. Future studies would benefit from greater standardization of assessment protocols and validation of a core outcome set across all participants.

Additional potentially important confounders, including prosthetic experience before enrollment, daily prosthesis use, social support, associated musculoskeletal injuries, and adherence to rehabilitation activities outside supervised sessions, were not systematically measured and may have influenced rehabilitation outcomes. Furthermore, exploratory analyses have limited generalizability because they were not externally validated and some analyses were not adjusted for multiple comparisons.

Future studies should include larger multicenter cohorts, more frequent psychiatric monitoring throughout rehabilitation, longer-term follow-up assessments at 6 and 12 months, and appropriate military comparison groups. Randomized studies integrating psychiatric interventions with rehabilitation programs are also needed to determine whether the observed associations can be modified and whether functional outcomes can be further improved.

## 5 Conclusions

The level of PTSD and depression symptoms at baseline was independently associated with how well and how quickly function recovered during 120 days of robotic neurorehabilitation after combat transtibial amputation. GLMM showed a significant interaction of “time × psychiatric group” for various functional, gait, and posturographic measures. This means that psychiatric status at baseline influenced not only the final outcome but also the dynamics of recovery itself.

Preliminary predictive analysis showed that there are certain threshold levels of symptoms at which the probability of a positive response to rehabilitation is markedly reduced, especially at high PCL-5 values. A simplified model with five variables (PCL-5, PHQ-9, VAS, baseline BBS, and TUG) showed stable results across multiple replicates. Cluster analysis also identified two distinct types of rehabilitation outcomes that differed in the level of psychiatric symptoms and the degree of functional recovery.

Overall, these results suggest that assessment of psychiatric status at the beginning of rehabilitation may be important in predicting recovery after combat amputation. However, due to limitations of the study (small sample size, observational design, and preliminary nature of the analysis), these findings need to be confirmed in larger, multicenter studies. It is also necessary to examine whether early psychiatric care can improve rehabilitation outcomes in the long term.

## Data Availability

The raw data supporting the conclusions of this article will be made available by the authors, without undue reservation.

## Supplementary material

Supplementary Figure S1 Individual rehabilitation trajectories by PCL-5 quartile (spaghetti plots). BBS (a) and TUG (b) trajectories for all 45 participants individually, coloured by PCL-5 quartile (Q1 < 30 to Q4 ≥ 64). Thin lines = individual patients; bold lines = quartile means.

Supplementary Figure S2 LOESS dose-response curves and retrospective power analysis. (a) LOESS-smoothed estimates of ΔBBS, Δ6MWT, and ΔFRT as continuous functions of baseline PCL-5. (b) Observed statistical power at *n* = 45 for primary outcomes.

Supplementary Figure S3 Posturographic heatmaps by PCL-5 group. Row-normalized median stabilometric values (Romberg Test; 12 parameters × 4 timepoints) for the PCL-5 < 33 (a) and PCL-5 ≥ 33 (b) groups.
