# Supplementary material for: Baseline PTSD and depressive symptoms predict multidimensional recovery after combat-related transtibial amputation: a prospective cohort study
Source: Front Rehabil Sci. 2026 Sep 10;7:1891994. doi: 10.3389/fresc.2026.1891994 (PMC13601325; doi:10.3389/fresc.2026.1891994)
Supplement: Supplementary file 1 [file Table1.docx]

**Supplementary Tables S1–S8**

Baseline PTSD Severity Is Associated With Attenuated Longitudinal Recovery Trajectories During Robotic Neurorehabilitation After Combat-Related Transtibial Amputation

Exploratory analyses (Supplementary Tables S6–S8) were not pre-specified, were not adjusted for multiple comparisons, and were not validated in independent samples. They should be interpreted with caution.

**Supplementary Table S1. Whole-Group Rehabilitation Outcomes (n = 45)**

Median [Q1–Q3] at Days 0, 14, 30, and 120 for all 46 outcome variables. p = Wilcoxon signed-rank (paired comparisons). Friedman p = Friedman test (repeated measures). Bold terracotta = p < 0.05.

| **Variable** | **Median [Q1–Q3] by Assessment Timepoint** | | | | **Wilcoxon Signed-Rank p** | | | | | **Friedman p** |
| --- | --- | --- | --- | --- | --- | --- | --- | --- | --- | --- |
|  | **Day 0** | **Day 14** | **Day 30** | **Day 120** | **D0→D14** | **D0→D30** | **D0→D120** | **D14→D30** | **D14→D120** |  |
| **Stabiloplatforma Alfa — Romberg Test** | | | | | | | | | | |
| **COP X-axis displacement, eyes open (cm)** | 2.24 (2.12–2.33) | 2.20 (2.10–2.29) | 2.17 (2.02–2.26) | 2.18 (1.98–2.25) | **<0.001** | **<0.001** | **<0.001** | **<0.001** | **<0.001** | **<0.001** |
| **COP X-axis displacement, eyes closed (cm)** | 2.74 (2.69–2.81) | 2.71 (2.55–2.78) | 2.69 (2.48–2.76) | 2.68 (2.40–2.77) | **<0.001** | **<0.001** | **<0.001** | **<0.001** | **<0.001** | **<0.001** |
| **COP Y-axis displacement, eyes open (cm)** | 2.84 (2.76–2.91) | 2.81 (2.65–2.87) | 2.79 (2.52–2.86) | 2.78 (2.40–2.87) | **<0.001** | **<0.001** | **<0.001** | **<0.001** | **<0.001** | **<0.001** |
| **COP Y-axis displacement, eyes closed (cm)** | 3.67 (3.61–3.76) | 3.65 (3.56–3.72) | 3.60 (3.18–3.71) | 3.60 (3.05–3.70) | **<0.001** | **<0.001** | **<0.001** | **<0.001** | **<0.001** | **<0.001** |
| **Mean velocity X-axis, eyes open (cm/s)** | 1.40 (1.28–1.51) | 1.28 (1.08–1.42) | 1.23 (0.87–1.36) | 1.23 (0.63–1.36) | **<0.001** | **<0.001** | **<0.001** | **<0.001** | **<0.001** | **<0.001** |
| **Mean velocity X-axis, eyes closed (cm/s)** | 3.18 (2.92–4.11) | 3.12 (2.90–3.52) | 2.98 (2.80–3.18) | 2.84 (2.07–3.15) | **<0.001** | **<0.001** | **<0.001** | **<0.001** | **<0.001** | **<0.001** |
| **Mean velocity Y-axis, eyes open (cm/s)** | 1.40 (1.30–1.50) | 1.28 (1.07–1.44) | 1.25 (0.92–1.42) | 1.25 (0.86–1.44) | **<0.001** | **<0.001** | **<0.001** | **<0.001** | **<0.001** | **<0.001** |
| **Mean velocity Y-axis, eyes closed (cm/s)** | 3.41 (2.88–3.77) | 3.05 (2.36–3.41) | 2.89 (2.20–3.42) | 2.72 (1.76–3.42) | **<0.001** | **<0.001** | **<0.001** | **<0.001** | **<0.001** | **<0.001** |
| **Area of oscillation, eyes open (cm²)** | 15.10 (14.60–15.70) | 14.80 (12.80–15.50) | 14.40 (10.20–15.50) | 14.50 (9.50–15.60) | **<0.001** | **<0.001** | **<0.001** | **<0.001** | **0.040** | **<0.001** |
| **Area of oscillation, eyes closed (cm²)** | 23.60 (22.80–24.50) | 22.70 (18.80–24.10) | 22.50 (15.80–24.30) | 22.70 (13.10–24.10) | **<0.001** | **<0.001** | **<0.001** | **<0.001** | **0.005** | **<0.001** |
| **COP path length, eyes open (cm)** | 36.60 (35.40–39.40) | 36.70 (34.60–39.00) | 35.10 (28.90–37.20) | 34.90 (26.20–37.10) | **<0.001** | **<0.001** | **<0.001** | **<0.001** | **0.031** | **<0.001** |
| **COP path length, eyes closed (cm)** | 89.50 (87.20–91.30) | 87.60 (74.80–91.60) | 87.30 (62.40–91.30) | 87.30 (41.90–91.50) | **<0.001** | **<0.001** | **<0.001** | **0.001** | 0.073 | **<0.001** |
| **Stabiloplatforma Alfa — Unterberger Test** | | | | | | | | | | |
| **Angle deviation per step (°)** | 6.60 (6.40–6.90) | 6.60 (6.10–7.00) | 6.30 (4.80–6.80) | 6.40 (4.16–6.90) | **<0.001** | **<0.001** | **<0.001** | **<0.001** | **0.049** | **<0.001** |
| **Movement amplitude (°)** | 15.50 (14.70–16.20) | 15.20 (13.80–16.00) | 14.80 (11.30–16.00) | 14.90 (8.10–16.10) | **<0.001** | **<0.001** | **0.001** | **<0.001** | **<0.001** | **<0.001** |
| **Stabiloplatforma Alfa — Movement Time Test** | | | | | | | | | | |
| **Movement time, left upper diagonal (ms)** | 4250.00 (4180.00–4460.00) | 4220.00 (4100.00–4440.00) | 4190.00 (4060.00–4400.00) | 4185.00 (3800.00–4365.00) | **<0.001** | **<0.001** | **<0.001** | **<0.001** | **0.016** | **<0.001** |
| **Movement time, right upper diagonal (ms)** | 6340.00 (6180.00–6633.00) | 6250.00 (6130.00–6540.00) | 6180.00 (6060.00–6440.00) | 6105.00 (5600.00–6385.00) | **<0.001** | **<0.001** | **<0.001** | **<0.001** | **0.008** | **<0.001** |
| **Movement time, left lower diagonal (ms)** | 5800.00 (5690.00–6100.00) | 5740.00 (5660.00–6000.00) | 5690.00 (5580.00–5860.00) | 5685.00 (5400.00–5875.00) | **<0.001** | **<0.001** | **<0.001** | **<0.001** | **0.005** | **<0.001** |
| **Movement time, right lower diagonal (ms)** | 4710.00 (4590.00–5100.00) | 4650.00 (4570.00–4950.00) | 4600.00 (4500.00–4840.00) | 4596.00 (4410.00–4760.00) | **<0.001** | **<0.001** | **<0.001** | **<0.001** | **0.002** | **<0.001** |
| **OMEGO — Balance & Force Assessment** | | | | | | | | | | |
| **Weight distribution, prosthetic side (%)** | 34.00 (34.00–35.00) | 35.00 (34.00–40.00) | 35.00 (34.00–46.00) | 35.00 (34.00–49.00) | **<0.001** | **<0.001** | **<0.001** | **0.009** | **0.002** | **0.003** |
| **Weight distribution, healthy side (%)** | 66.00 (65.00–66.00) | 65.00 (60.00–66.00) | 65.00 (54.00–66.00) | 65.00 (51.00–66.00) | **<0.001** | **<0.001** | **<0.001** | **0.009** | **0.002** | **0.003** |
| **Proprioception difference, mean (°)** | 71.00 (67.00–74.00) | 57.00 (49.00–70.00) | 44.00 (36.00–71.00) | 36.00 (30.00–72.00) | **<0.001** | **<0.001** | **<0.001** | **<0.001** | **<0.001** | **0.045** |
| **Force sensation, healthy limb (kg)** | 71.00 (69.50–74.80) | 70.50 (69.30–72.60) | 70.00 (66.90–71.40) | 69.20 (64.60–70.40) | **<0.001** | **<0.001** | **<0.001** | **<0.001** | **<0.001** | **<0.001** |
| **Force sensation, affected limb (kg)** | 38.90 (34.30–40.10) | 41.10 (39.80–46.90) | 45.70 (40.20–52.50) | 48.90 (40.30–55.50) | **<0.001** | **<0.001** | **<0.001** | **0.040** | **0.005** | **<0.001** |
| **STRIDE ONE System** | | | | | | | | | | |
| **Smoothness Index (a.u.)** | 16.70 (15.40–19.90) | 15.50 (14.50–16.40) | 14.60 (12.80–15.70) | 13.80 (10.90–15.50) | **<0.001** | **<0.001** | **<0.001** | **<0.001** | **0.002** | **<0.001** |
| **Symmetry Index (%)** | 59.40 (53.90–64.70) | 60.50 (56.70–75.40) | 80.90 (57.70–86.80) | 85.10 (56.50–91.90) | **<0.001** | **<0.001** | **<0.001** | **0.004** | **<0.001** | 0.063 |
| **Roll-off quality, healthy limb (%)** | 86.00 (84.00–90.00) | 90.00 (87.00–93.00) | 89.00 (85.00–94.00) | 90.00 (86.00–96.00) | **<0.001** | **0.032** | **<0.001** | **0.002** | 0.837 | **<0.001** |
| **Roll-off quality, affected limb (%)** | 49.00 (46.00–59.00) | 64.00 (49.00–84.00) | 63.00 (48.00–88.00) | 64.00 (48.00–89.00) | **<0.001** | **<0.001** | **<0.001** | 0.313 | **0.016** | **<0.001** |
| **Stance ratio, healthy limb (%)** | 55.00 (52.00–60.00) | 58.00 (55.00–62.00) | 57.00 (54.00–61.00) | 58.00 (55.00–62.00) | **<0.001** | **0.002** | **0.005** | 0.182 | 0.541 | **<0.001** |
| **Stance ratio, affected limb (%)** | 79.00 (77.00–82.00) | 76.00 (72.00–79.00) | 77.00 (72.00–79.00) | 76.00 (72.00–78.00) | **0.001** | **0.011** | **<0.001** | **0.044** | 0.587 | **<0.001** |
| **Step duration, healthy limb (s)** | 2.09 (1.95–2.15) | 2.04 (1.72–2.11) | 2.05 (1.19–2.10) | 2.06 (1.20–2.12) | 0.104 | **<0.001** | 0.097 | **<0.001** | **0.010** | **<0.001** |
| **Step duration, affected limb (s)** | 2.39 (2.31–2.58) | 2.34 (2.04–2.39) | 2.33 (1.64–2.39) | 2.34 (1.40–2.41) | **0.003** | **0.003** | **0.002** | **0.013** | **0.011** | **0.006** |
| **BALO Platform** | | | | | | | | | | |
| **Anterior-posterior stability index (a.u.)** | 1240.00 (1225.00–1265.00) | 1285.00 (1245.00–2475.00) | 1265.00 (1228.00–4995.00) | 1290.00 (1238.00–7420.00) | **<0.001** | **<0.001** | **<0.001** | **0.020** | **<0.001** | **<0.001** |
| **Dynamic mediolateral stability index (a.u.)** | 1250.00 (1235.00–1265.00) | 1280.00 (1235.00–2690.00) | 1268.00 (1228.00–5230.00) | 1280.00 (1245.00–7680.00) | **<0.001** | **0.001** | **<0.001** | **0.024** | **<0.001** | **<0.001** |
| **Lateral stability index (a.u.)** | 1255.00 (1232.00–1270.00) | 1280.00 (1240.00–2860.00) | 1270.00 (1230.00–5515.00) | 1290.00 (1240.00–7880.00) | **<0.001** | **<0.001** | **<0.001** | **0.005** | **<0.001** | **<0.001** |
| **Functional Tests & Clinical Scales** | | | | | | | | | | |
| **Functional Reach Test (cm)** | 11.00 (9.00–12.00) | 13.00 (10.00–18.00) | 13.00 (11.00–19.00) | 13.00 (10.00–22.00) | **<0.001** | **<0.001** | **<0.001** | 0.121 | **<0.001** | **<0.001** |
| **Berg Balance Scale (pts)** | 32.00 (26.00–35.00) | 35.00 (33.00–36.00) | 37.00 (34.00–45.00) | 36.00 (33.00–45.00) | **<0.001** | **<0.001** | **<0.001** | **0.003** | **<0.001** | **<0.001** |
| **Timed Up and Go (s)** | 31.00 (29.00–37.00) | 28.00 (22.00–30.00) | 28.00 (13.00–31.00) | 28.00 (13.00–30.00) | **<0.001** | **0.017** | **<0.001** | 0.167 | **0.007** | **<0.001** |
| **Amputee Mobility Predictor (pts)** | 23.00 (22.00–25.00) | 26.00 (23.00–32.00) | 25.00 (23.00–38.00) | 25.00 (23.00–44.00) | **<0.001** | **<0.001** | **<0.001** | **0.003** | **0.003** | **<0.001** |
| **Four Square Step Test (s)** | 23.00 (18.00–25.00) | 17.00 (15.00–22.00) | 17.00 (13.00–23.00) | 16.00 (12.00–22.00) | **<0.001** | **0.003** | **<0.001** | 0.646 | 0.192 | **<0.001** |
| **10-Meter Walk Test (m/s)** | 0.41 (0.37–0.45) | 0.45 (0.41–0.52) | 0.50 (0.43–0.86) | 0.49 (0.42–1.00) | **<0.001** | **<0.001** | **<0.001** | **<0.001** | **<0.001** | **<0.001** |
| **2-Minute Walk Test (m)** | 55.00 (52.00–61.00) | 60.00 (56.00–70.00) | 62.00 (54.00–88.00) | 61.00 (56.00–132.00) | **<0.001** | **<0.001** | **<0.001** | **0.002** | **<0.001** | **<0.001** |
| **6-Minute Walk Test (m)** | 165.00 (143.00–204.00) | 182.00 (167.00–215.00) | 179.00 (164.00–287.00) | 176.00 (144.00–410.00) | **<0.001** | **<0.001** | **<0.001** | **0.007** | **<0.001** | **<0.001** |
| **Activities-specific Balance Confidence Scale (%)** | 44.00 (36.00–50.00) | 45.00 (43.00–56.00) | 49.00 (42.00–58.00) | 51.00 (40.00–73.00) | **<0.001** | **0.001** | **<0.001** | 0.313 | **<0.001** | **<0.001** |
| **Visual Analog Scale — pain (pts)** | 4.00 (3.00–5.00) | 3.00 (2.00–4.00) | 2.00 (1.00–3.00) | 2.00 (1.00–3.00) | **<0.001** | **<0.001** | **<0.001** | **<0.001** | **<0.001** | **<0.001** |
| **PTSD Checklist (PCL-5) (pts)** | 57.00 (30.00–67.00) | — | — | 32.00 (21.00–55.00) | — | — | **<0.001** | — | — | — |
| **Patient Health Questionnaire (PHQ-9) (pts)** | 15.00 (14.00–18.00) | — | — | 12.00 (9.00–14.00) | — | — | **<0.001** | — | — | — |

**Supplementary Table S2. Stratification by VAS Level (Pain Severity)**

Group 0: VAS 0–3 (mild pain) (n=20) vs Group 1: VAS 4–6 (moderate pain) (n=25). Within-group p = Wilcoxon signed-rank (D0→D120). Between-group p = Mann–Whitney U at each timepoint. Bold terracotta = p < 0.05.

| **Variable** | **Group 0: VAS 0–3 (mild pain) (n=20)** | | | | **Group 1: VAS 4–6 (moderate pain) (n=25)** | | | | **Within-group p (D0→D120)** | | **Between-group p (Mann–Whitney U)** | | | |
| --- | --- | --- | --- | --- | --- | --- | --- | --- | --- | --- | --- | --- | --- | --- |
|  | **D0** | **D14** | **D30** | **D120** | **D0** | **D14** | **D30** | **D120** | **p G0** | **p G1** | **D0** | **D14** | **D30** | **D120** |
| **Stabiloplatforma Alfa — Romberg Test** | | | | | | | | | | | | | | |
| **COP X-axis displacement, eyes open (cm)** | 2.35 (2.18–2.50) | 2.26 (1.80–2.38) | 2.16 (1.66–2.30) | 1.83 (1.52–2.26) | 2.18 (2.12–2.26) | 2.17 (2.10–2.23) | 2.17 (2.08–2.24) | 2.18 (2.09–2.25) | **<0.001** | **0.003** | **0.013** | 0.320 | 0.639 | 0.188 |
| **COP X-axis displacement, eyes closed (cm)** | 2.76 (2.72–2.85) | 2.52 (2.44–2.78) | 2.13 (2.08–2.71) | 2.09 (1.98–2.70) | 2.74 (2.69–2.77) | 2.71 (2.69–2.76) | 2.71 (2.67–2.76) | 2.72 (2.68–2.77) | **<0.001** | **0.003** | 0.200 | 0.066 | **0.026** | **0.004** |
| **COP Y-axis displacement, eyes open (cm)** | 2.84 (2.76–2.92) | 2.63 (2.36–2.87) | 2.18 (2.12–2.81) | 2.08 (1.96–2.81) | 2.84 (2.77–2.88) | 2.81 (2.76–2.86) | 2.80 (2.74–2.86) | 2.80 (2.75–2.87) | **<0.001** | **0.002** | 0.973 | 0.080 | **0.031** | **0.008** |
| **COP Y-axis displacement, eyes closed (cm)** | 3.71 (3.63–3.79) | 3.64 (3.24–3.71) | 3.18 (3.03–3.62) | 2.98 (2.75–3.60) | 3.67 (3.61–3.73) | 3.67 (3.60–3.72) | 3.64 (3.59–3.74) | 3.64 (3.58–3.75) | **<0.001** | **0.003** | 0.493 | 0.128 | **0.003** | **0.002** |
| **Mean velocity X-axis, eyes open (cm/s)** | 1.46 (1.35–1.52) | 1.29 (1.07–1.42) | 1.18 (0.77–1.32) | 0.93 (0.58–1.30) | 1.35 (1.18–1.45) | 1.27 (1.10–1.43) | 1.24 (1.09–1.36) | 1.25 (0.95–1.37) | **<0.001** | **<0.001** | 0.089 | 0.927 | 0.411 | 0.140 |
| **Mean velocity X-axis, eyes closed (cm/s)** | 3.18 (2.95–4.30) | 3.11 (2.90–3.35) | 2.93 (2.79–3.12) | 2.16 (1.94–2.87) | 3.22 (2.92–4.03) | 3.15 (2.91–3.68) | 3.08 (2.87–3.21) | 2.99 (2.17–3.26) | **<0.001** | **<0.001** | 0.486 | 0.500 | 0.213 | **0.020** |
| **Mean velocity Y-axis, eyes open (cm/s)** | 1.42 (1.34–1.51) | 1.27 (1.09–1.39) | 1.21 (0.91–1.31) | 1.10 (0.82–1.29) | 1.37 (1.22–1.50) | 1.32 (1.07–1.47) | 1.30 (1.13–1.43) | 1.32 (1.14–1.46) | **<0.001** | **0.002** | 0.391 | 0.385 | 0.209 | **0.046** |
| **Mean velocity Y-axis, eyes closed (cm/s)** | 3.27 (2.74–3.89) | 2.71 (2.38–3.15) | 2.64 (2.20–3.10) | 2.27 (1.59–2.77) | 3.48 (2.95–3.73) | 3.22 (2.36–3.59) | 3.15 (2.49–3.56) | 3.16 (2.09–3.59) | **<0.001** | **0.002** | 0.882 | 0.081 | 0.061 | **0.027** |
| **Area of oscillation, eyes open (cm²)** | 14.95 (14.47–15.72) | 14.75 (13.12–15.07) | 14.30 (10.75–15.03) | 14.45 (10.03–15.15) | 15.20 (14.70–15.70) | 15.10 (12.80–15.70) | 14.50 (10.20–16.10) | 14.50 (9.50–15.70) | **0.003** | **0.009** | 0.784 | 0.437 | 0.451 | 0.615 |
| **Area of oscillation, eyes closed (cm²)** | 23.65 (22.73–24.57) | 22.35 (19.10–23.57) | 22.20 (15.80–23.82) | 22.35 (13.03–23.95) | 23.60 (22.80–24.40) | 22.80 (18.80–24.50) | 22.60 (15.80–25.10) | 22.80 (13.10–24.10) | **0.002** | **0.003** | 0.936 | 0.486 | 0.530 | 0.648 |
| **COP path length, eyes open (cm)** | 36.00 (34.90–38.35) | 35.80 (34.60–38.62) | 34.90 (31.55–36.07) | 34.80 (28.45–36.27) | 36.90 (35.70–41.90) | 36.80 (34.90–39.20) | 35.10 (28.90–39.10) | 35.00 (26.20–37.40) | **0.003** | **0.008** | 0.437 | 0.472 | 0.486 | 0.560 |
| **COP path length, eyes closed (cm)** | 89.40 (87.17–91.55) | 86.90 (74.65–89.80) | 86.50 (62.02–89.50) | 86.90 (39.27–89.90) | 89.50 (87.90–91.10) | 87.90 (75.00–93.20) | 87.60 (66.50–92.90) | 87.70 (44.20–93.30) | **0.002** | **0.032** | 0.766 | 0.500 | 0.424 | 0.367 |
| **Stabiloplatforma Alfa — Unterberger Test** | | | | | | | | | | | | | | |
| **Angle deviation per step (°)** | 6.55 (6.28–6.90) | 6.45 (5.62–6.83) | 6.30 (4.20–6.72) | 6.35 (3.65–6.80) | 6.70 (6.50–7.10) | 6.70 (6.30–7.00) | 6.40 (5.20–7.00) | 6.50 (4.53–7.10) | **0.012** | **0.015** | 0.303 | 0.200 | 0.391 | 0.355 |
| **Movement amplitude (°)** | 15.25 (14.55–16.12) | 14.95 (12.95–15.70) | 14.75 (10.55–15.53) | 14.80 (7.33–15.62) | 15.50 (15.00–16.20) | 15.30 (14.10–16.10) | 14.90 (11.50–17.20) | 15.00 (9.00–17.70) | **0.008** | **0.034** | 0.464 | 0.320 | 0.367 | 0.355 |
| **Stabiloplatforma Alfa — Movement Time Test** | | | | | | | | | | | | | | |
| **Movement time, left upper diagonal (ms)** | 4220.00 (4105.00–4392.50) | 4185.00 (4067.50–4380.00) | 4170.00 (4032.50–4365.00) | 4055.00 (3532.50–4210.00) | 4310.00 (4220.00–4567.00) | 4240.00 (4180.00–4450.00) | 4200.00 (4090.00–4400.00) | 4200.00 (4010.00–4500.00) | **<0.001** | **0.038** | 0.235 | 0.239 | 0.417 | **0.049** |
| **Movement time, right upper diagonal (ms)** | 6280.00 (6110.00–6593.25) | 6215.00 (6095.00–6472.50) | 6180.00 (6015.00–6455.00) | 6047.50 (5222.50–6212.50) | 6370.00 (6230.00–6905.00) | 6280.00 (6180.00–6570.00) | 6230.00 (6090.00–6410.00) | 6245.00 (5900.00–6400.00) | **<0.001** | **0.005** | 0.361 | 0.355 | 0.508 | 0.100 |
| **Movement time, left lower diagonal (ms)** | 5755.00 (5617.50–6010.00) | 5705.00 (5597.50–5975.00) | 5685.00 (5576.25–5832.50) | 5640.00 (5390.00–5771.25) | 5830.00 (5720.00–6100.00) | 5800.00 (5680.00–6000.00) | 5700.00 (5580.00–5860.00) | 5735.00 (5400.00–5900.00) | **0.001** | **0.007** | 0.304 | 0.349 | 0.732 | 0.417 |
| **Movement time, right lower diagonal (ms)** | 4660.00 (4550.00–4935.00) | 4605.00 (4538.75–4875.00) | 4590.00 (4522.50–4812.50) | 4565.00 (4400.00–4733.75) | 4730.00 (4620.00–5100.00) | 4680.00 (4580.00–4950.00) | 4620.00 (4500.00–4840.00) | 4635.00 (4448.00–4765.00) | **<0.001** | **0.007** | 0.320 | 0.404 | 0.740 | 0.398 |
| **OMEGO — Balance & Force Assessment** | | | | | | | | | | | | | | |
| **Weight distribution, prosthetic side (%)** | 34.50 (34.00–35.00) | 35.00 (34.00–40.50) | 35.00 (34.00–46.00) | 35.00 (34.75–49.00) | 34.00 (33.00–35.00) | 35.00 (33.00–40.00) | 35.00 (34.00–45.00) | 35.00 (33.00–49.00) | **0.012** | **0.015** | 0.729 | 0.474 | 0.432 | 0.488 |
| **Weight distribution, healthy side (%)** | 65.50 (65.00–66.00) | 65.00 (59.50–66.00) | 65.00 (54.00–66.00) | 65.00 (51.00–65.25) | 66.00 (65.00–67.00) | 65.00 (60.00–67.00) | 65.00 (55.00–66.00) | 65.00 (51.00–67.00) | **0.012** | **0.015** | 0.729 | 0.474 | 0.432 | 0.488 |
| **Proprioception difference, mean (°)** | 72.00 (67.50–75.50) | 53.50 (48.25–68.50) | 41.00 (35.50–68.75) | 33.50 (29.50–69.75) | 70.00 (67.00–73.00) | 62.00 (50.00–71.00) | 58.00 (37.00–72.00) | 54.00 (30.00–73.00) | **0.002** | **0.009** | 0.537 | 0.391 | 0.373 | 0.379 |
| **Force sensation, healthy limb (kg)** | 71.50 (69.72–76.15) | 71.05 (68.83–73.07) | 69.85 (65.35–71.10) | 68.35 (62.90–70.50) | 71.00 (69.40–73.20) | 70.40 (69.30–72.60) | 70.10 (67.70–71.80) | 69.60 (65.10–70.40) | **0.001** | **0.002** | 0.623 | 0.802 | 0.656 | 0.493 |
| **Force sensation, affected limb (kg)** | 38.30 (34.90–40.15) | 44.25 (40.35–49.20) | 47.75 (40.85–52.65) | 50.95 (40.95–55.92) | 39.30 (33.10–40.00) | 40.90 (39.60–43.60) | 41.70 (39.90–50.80) | 41.80 (40.00–53.60) | **0.001** | **0.002** | 0.664 | 0.095 | 0.268 | 0.239 |
| **STRIDE ONE System** | | | | | | | | | | | | | | |
| **Smoothness Index (a.u.)** | 16.40 (15.28–19.77) | 15.10 (12.25–16.32) | 14.45 (11.88–15.53) | 13.20 (10.17–14.70) | 16.80 (15.60–19.90) | 15.80 (14.70–16.40) | 15.10 (14.10–15.70) | 14.90 (12.10–17.20) | **<0.001** | **0.014** | 0.648 | 0.361 | 0.278 | 0.089 |
| **Symmetry Index (%)** | 58.60 (53.80–69.47) | 61.25 (55.93–80.57) | 82.35 (59.95–87.35) | 86.60 (58.88–92.72) | 60.10 (54.10–63.20) | 59.70 (57.40–71.70) | 70.10 (56.90–84.20) | 74.00 (55.70–89.10) | **<0.001** | **0.008** | 0.900 | 0.723 | 0.309 | 0.283 |
| **Roll-off quality, healthy limb (%)** | 86.00 (82.00–89.00) | 91.50 (88.50–96.25) | 92.50 (87.50–96.25) | 95.00 (88.50–97.25) | 87.00 (85.00–90.00) | 88.00 (85.00–90.00) | 86.00 (83.00–90.00) | 87.00 (84.00–91.00) | **<0.001** | 0.057 | 0.376 | **0.041** | **0.014** | **0.015** |
| **Roll-off quality, affected limb (%)** | 49.00 (43.50–59.00) | 81.50 (49.75–88.25) | 85.50 (48.75–92.25) | 87.50 (49.75–91.00) | 49.00 (46.00–59.00) | 51.00 (48.00–68.00) | 50.00 (47.00–75.00) | 51.00 (47.00–83.00) | **<0.001** | **0.002** | 0.882 | 0.125 | 0.086 | 0.097 |
| **Stance ratio, healthy limb (%)** | 54.00 (48.75–58.50) | 58.50 (55.75–62.25) | 57.00 (55.50–60.25) | 58.00 (55.00–61.25) | 56.00 (54.00–62.00) | 58.00 (55.00–62.00) | 57.00 (54.00–63.00) | 57.00 (55.00–63.00) | 0.131 | **0.011** | 0.216 | 0.810 | 0.846 | 0.881 |
| **Stance ratio, affected limb (%)** | 78.50 (75.00–81.25) | 74.50 (71.00–77.25) | 72.50 (70.75–76.25) | 73.50 (71.00–76.00) | 79.00 (77.00–83.00) | 77.00 (76.00–79.00) | 78.00 (76.00–80.00) | 77.00 (75.00–79.00) | **<0.001** | 0.076 | 0.335 | **0.038** | **0.005** | **0.009** |
| **Step duration, healthy limb (s)** | 2.09 (1.94–2.20) | 1.94 (1.67–2.10) | 1.23 (1.15–2.07) | 1.21 (1.12–2.08) | 2.09 (1.98–2.12) | 2.06 (1.88–2.13) | 2.08 (1.71–2.12) | 2.10 (1.72–2.16) | **0.002** | 0.148 | 0.731 | 0.107 | **0.008** | **0.004** |
| **Step duration, affected limb (s)** | 2.42 (2.33–2.65) | 2.19 (1.86–2.38) | 1.71 (1.49–2.37) | 1.42 (1.35–2.38) | 2.38 (2.28–2.44) | 2.37 (2.25–2.40) | 2.39 (2.02–2.45) | 2.38 (2.03–2.44) | **0.001** | 0.386 | 0.221 | 0.092 | **0.018** | **0.012** |
| **BALO Platform** | | | | | | | | | | | | | | |
| **Anterior-posterior stability index (a.u.)** | 1240.00 (1223.25–1266.25) | 2405.00 (1267.50–2560.00) | 3675.00 (1254.75–5000.00) | 5650.00 (1275.00–7435.00) | 1245.00 (1228.00–1265.00) | 1260.00 (1230.00–2430.00) | 1255.00 (1180.00–4995.00) | 1270.00 (995.00–7420.00) | **<0.001** | 0.054 | 0.855 | **0.036** | 0.189 | 0.174 |
| **Dynamic mediolateral stability index (a.u.)** | 1250.00 (1235.00–1265.75) | 1917.50 (1242.00–2698.75) | 2811.00 (1238.00–5231.25) | 3869.00 (1253.75–7687.50) | 1248.00 (1238.00–1260.00) | 1268.00 (1220.00–2680.00) | 1260.00 (1160.00–5230.00) | 1275.00 (975.00–7680.00) | **0.002** | 0.054 | 0.882 | 0.304 | 0.404 | 0.479 |
| **Lateral stability index (a.u.)** | 1252.50 (1231.50–1270.00) | 1812.50 (1240.00–2868.75) | 2761.00 (1243.50–5488.75) | 3807.50 (1244.75–7857.50) | 1255.00 (1235.00–1268.00) | 1272.00 (1238.00–2860.00) | 1268.00 (1230.00–5515.00) | 1280.00 (1240.00–7880.00) | **0.001** | **0.018** | 0.927 | 0.672 | 0.681 | 0.640 |
| **Functional Tests & Clinical Scales** | | | | | | | | | | | | | | |
| **Functional Reach Test (cm)** | 11.00 (9.00–12.25) | 16.50 (12.75–19.25) | 17.50 (12.75–21.25) | 20.50 (12.75–24.25) | 11.00 (9.00–12.00) | 11.00 (10.00–13.00) | 12.00 (9.00–14.00) | 11.00 (10.00–13.00) | **<0.001** | 0.136 | 0.547 | **0.003** | **0.004** | **0.005** |
| **Berg Balance Scale (pts)** | 27.50 (24.00–32.00) | 34.00 (32.00–37.00) | 43.00 (34.75–48.25) | 43.50 (34.75–48.25) | 34.00 (32.00–37.00) | 35.00 (34.00–36.00) | 35.00 (34.00–38.00) | 35.00 (33.00–39.00) | **<0.001** | 0.117 | **0.006** | 0.311 | **0.046** | **0.029** |
| **Timed Up and Go (s)** | 31.00 (28.50–36.25) | 23.50 (19.75–27.50) | 14.50 (11.00–28.50) | 14.50 (11.00–27.50) | 31.00 (29.00–38.00) | 30.00 (27.00–31.00) | 31.00 (27.00–32.00) | 30.00 (27.00–31.00) | **<0.001** | 0.155 | 0.854 | **0.004** | **0.004** | **0.003** |
| **Amputee Mobility Predictor (pts)** | 23.00 (21.75–25.00) | 30.50 (23.75–33.25) | 36.50 (23.75–39.00) | 43.00 (24.00–47.00) | 23.00 (22.00–25.00) | 24.00 (23.00–27.00) | 24.00 (23.00–25.00) | 24.00 (23.00–26.00) | **<0.001** | 0.101 | 0.360 | 0.077 | 0.073 | 0.060 |
| **Four Square Step Test (s)** | 22.50 (18.00–25.00) | 16.00 (14.00–17.25) | 14.50 (12.00–18.00) | 12.50 (10.75–17.00) | 23.00 (20.00–24.00) | 21.00 (16.00–22.00) | 22.00 (15.00–23.00) | 21.00 (14.00–22.00) | **<0.001** | **0.036** | 0.899 | **0.015** | **0.004** | **0.005** |
| **10-Meter Walk Test (m/s)** | 0.41 (0.37–0.47) | 0.46 (0.41–0.52) | 0.82 (0.43–0.89) | 0.94 (0.42–1.08) | 0.41 (0.40–0.45) | 0.42 (0.41–0.52) | 0.44 (0.42–0.66) | 0.43 (0.41–0.82) | **<0.001** | **<0.001** | 0.654 | 0.536 | 0.130 | 0.122 |
| **2-Minute Walk Test (m)** | 52.00 (43.75–57.25) | 63.50 (56.00–72.75) | 82.50 (57.00–98.25) | 122.00 (56.00–135.25) | 58.00 (52.00–61.00) | 59.00 (55.00–64.00) | 57.00 (54.00–69.00) | 56.00 (53.00–71.00) | **<0.001** | **0.016** | 0.084 | 0.153 | **0.045** | **0.029** |
| **6-Minute Walk Test (m)** | 165.00 (143.00–206.25) | 208.50 (169.25–234.75) | 268.50 (168.00–310.50) | 394.50 (170.50–450.75) | 165.00 (160.00–198.00) | 175.00 (162.00–195.00) | 168.00 (160.00–212.00) | 166.00 (144.00–218.00) | **<0.001** | **0.016** | 0.818 | 0.112 | **0.041** | **0.018** |
| **Activities-specific Balance Confidence Scale (%)** | 42.00 (35.25–50.50) | 49.00 (42.25–56.00) | 55.00 (42.00–63.00) | 64.00 (43.00–76.50) | 44.00 (42.00–50.00) | 45.00 (43.00–51.00) | 44.00 (40.00–55.00) | 43.00 (37.00–56.00) | **<0.001** | **0.014** | 0.236 | 0.713 | 0.180 | 0.062 |
| **Visual Analog Scale — pain (pts)** | 2.50 (2.00–3.00) | 2.00 (2.00–2.00) | 1.50 (1.00–2.00) | 1.00 (1.00–2.00) | 5.00 (4.00–6.00) | 4.00 (3.00–5.00) | 3.00 (2.00–4.00) | 3.00 (2.00–4.00) | **0.004** | **<0.001** | **<0.001** | **<0.001** | **<0.001** | **<0.001** |
| **PTSD Checklist (PCL-5) (pts)** | 29.50 (28.00–31.25) | — | — | 21.00 (18.50–23.25) | 66.00 (60.00–69.00) | — | — | 54.00 (35.00–58.00) | **<0.001** | **<0.001** | **<0.001** | — | — | **<0.001** |
| **Patient Health Questionnaire (PHQ-9) (pts)** | 13.00 (11.75–14.00) | — | — | 9.50 (8.00–12.00) | 18.00 (16.00–19.00) | — | — | 13.00 (12.00–15.00) | **<0.001** | **<0.001** | **<0.001** | — | — | **<0.001** |

**Supplementary Table S3. Stratification by PCL-5 Level (PTSD Severity)**

Group 0: PCL-5 ≤33 (sub-threshold) (n=20) vs Group 1: PCL-5 >33 (probable PTSD) (n=25). Within-group p = Wilcoxon signed-rank (D0→D120). Between-group p = Mann–Whitney U at each timepoint. Bold terracotta = p < 0.05.

| **Variable** | **Group 0: PCL-5 < 33 (sub-threshold) (n=20)** | | | | **Group 1: PCL-5 >33 (probable PTSD) (n=25)** | | | | **Within-group p (D0→D120)** | | **Between-group p (Mann–Whitney U)** | | | |
| --- | --- | --- | --- | --- | --- | --- | --- | --- | --- | --- | --- | --- | --- | --- |
|  | **D0** | **D14** | **D30** | **D120** | **D0** | **D14** | **D30** | **D120** | **p G0** | **p G1** | **D0** | **D14** | **D30** | **D120** |
| **Stabiloplatforma Alfa — Romberg Test** | | | | | | | | | | | | | | |
| **COP X-axis displacement, eyes open (cm)** | 2.35 (2.14–2.50) | 2.25 (2.03–2.38) | 2.16 (1.73–2.30) | 1.98 (1.54–2.30) | 2.18 (2.12–2.26) | 2.18 (2.10–2.23) | 2.17 (2.08–2.24) | 2.18 (2.09–2.25) | **0.003** | **<0.001** | **0.026** | 0.355 | 0.873 | 0.450 |
| **COP X-axis displacement, eyes closed (cm)** | 2.74 (2.71–2.81) | 2.62 (2.44–2.76) | 2.40 (2.08–2.71) | 2.35 (1.98–2.70) | 2.74 (2.69–2.81) | 2.73 (2.68–2.78) | 2.71 (2.67–2.76) | 2.72 (2.68–2.77) | **0.003** | **<0.001** | 0.973 | 0.056 | **0.048** | **0.011** |
| **COP Y-axis displacement, eyes open (cm)** | 2.82 (2.75–2.87) | 2.71 (2.36–2.85) | 2.46 (2.12–2.81) | 2.36 (1.96–2.81) | 2.85 (2.77–2.91) | 2.82 (2.76–2.88) | 2.80 (2.74–2.86) | 2.80 (2.74–2.87) | **0.003** | **<0.001** | 0.212 | 0.058 | 0.055 | **0.022** |
| **COP Y-axis displacement, eyes closed (cm)** | 3.66 (3.58–3.75) | 3.60 (3.24–3.69) | 3.35 (3.03–3.62) | 3.19 (2.75–3.60) | 3.69 (3.61–3.79) | 3.67 (3.62–3.74) | 3.64 (3.59–3.74) | 3.64 (3.58–3.75) | **0.002** | **<0.001** | 0.471 | **0.043** | **0.007** | **0.005** |
| **Mean velocity X-axis, eyes open (cm/s)** | 1.48 (1.35–1.53) | 1.31 (1.11–1.44) | 1.18 (0.82–1.31) | 0.97 (0.58–1.30) | 1.33 (1.18–1.44) | 1.26 (1.03–1.40) | 1.24 (1.02–1.38) | 1.25 (0.88–1.39) | **<0.001** | **0.004** | **0.020** | 0.500 | 0.479 | 0.131 |
| **Mean velocity X-axis, eyes closed (cm/s)** | 3.18 (3.01–4.37) | 3.12 (2.90–3.41) | 2.95 (2.81–3.09) | 2.16 (1.98–2.87) | 3.22 (2.92–3.98) | 3.12 (2.91–3.68) | 3.08 (2.75–3.29) | 2.99 (2.17–3.47) | **<0.001** | **0.004** | 0.326 | 0.615 | 0.235 | **0.016** |
| **Mean velocity Y-axis, eyes open (cm/s)** | 1.43 (1.35–1.56) | 1.27 (1.12–1.40) | 1.24 (0.94–1.38) | 1.16 (0.85–1.37) | 1.34 (1.22–1.47) | 1.31 (1.07–1.45) | 1.29 (0.92–1.43) | 1.30 (1.11–1.46) | **<0.001** | **0.010** | 0.105 | 0.873 | 0.479 | 0.147 |
| **Mean velocity Y-axis, eyes closed (cm/s)** | 3.35 (2.74–3.77) | 2.69 (2.36–3.15) | 2.64 (2.18–3.10) | 2.27 (1.58–2.77) | 3.45 (2.95–3.73) | 3.22 (2.72–3.59) | 3.15 (2.51–3.60) | 3.16 (2.09–3.61) | **<0.001** | **0.007** | 0.873 | 0.064 | **0.034** | **0.011** |
| **Area of oscillation, eyes open (cm²)** | 15.00 (14.40–15.72) | 14.65 (12.12–15.33) | 14.15 (9.85–15.43) | 14.15 (8.88–15.62) | 15.10 (14.70–15.70) | 14.90 (13.40–15.50) | 14.60 (11.00–15.60) | 14.60 (10.40–15.50) | **0.006** | **0.005** | 0.689 | 0.444 | 0.424 | 0.552 |
| **Area of oscillation, eyes closed (cm²)** | 23.95 (22.40–24.57) | 21.95 (18.38–23.85) | 21.80 (15.80–24.27) | 21.75 (12.78–24.45) | 23.50 (22.90–24.40) | 22.80 (19.30–24.30) | 22.60 (15.80–24.30) | 22.80 (13.30–24.00) | **0.005** | **0.002** | 0.837 | 0.431 | 0.493 | 0.576 |
| **COP path length, eyes open (cm)** | 36.90 (34.90–38.35) | 36.45 (33.60–39.02) | 34.60 (26.82–37.02) | 34.40 (24.62–37.25) | 36.20 (35.70–41.90) | 36.70 (35.20–38.90) | 35.10 (31.40–37.20) | 35.30 (27.50–36.90) | **0.006** | **0.005** | 0.775 | 0.648 | 0.451 | 0.508 |
| **COP path length, eyes closed (cm)** | 89.90 (87.05–91.55) | 86.70 (73.75–91.17) | 86.20 (60.73–90.85) | 86.30 (38.48–91.20) | 89.10 (88.00–91.10) | 88.00 (77.60–91.60) | 87.70 (70.10–91.30) | 88.00 (46.30–91.70) | **0.005** | **0.020** | 0.918 | 0.444 | 0.373 | 0.299 |
| **Stabiloplatforma Alfa — Unterberger Test** | | | | | | | | | | | | | | |
| **Angle deviation per step (°)** | 6.65 (6.28–6.90) | 6.45 (5.40–6.90) | 6.25 (4.05–6.83) | 6.25 (3.57–6.83) | 6.60 (6.50–7.10) | 6.70 (6.30–7.00) | 6.40 (5.30–6.80) | 6.50 (4.67–6.90) | **0.011** | **0.018** | 0.397 | 0.272 | 0.385 | 0.349 |
| **Movement amplitude (°)** | 15.45 (14.55–16.12) | 14.80 (11.97–15.77) | 14.65 (10.03–15.97) | 14.60 (7.10–16.07) | 15.50 (15.00–16.20) | 15.30 (14.60–16.00) | 15.00 (12.00–16.00) | 15.10 (9.20–16.10) | **0.018** | **0.022** | 0.639 | 0.361 | 0.373 | 0.349 |
| **Stabiloplatforma Alfa — Movement Time Test** | | | | | | | | | | | | | | |
| **Movement time, left upper diagonal (ms)** | 4270.00 (4105.00–4571.25) | 4190.00 (4067.50–4475.00) | 4150.00 (4032.50–4365.00) | 3970.00 (3250.00–4232.50) | 4250.00 (4200.00–4460.00) | 4230.00 (4180.00–4350.00) | 4210.00 (4090.00–4400.00) | 4200.00 (4075.00–4500.00) | **<0.001** | **0.033** | 0.560 | 0.451 | 0.361 | **0.028** |
| **Movement time, right upper diagonal (ms)** | 6360.00 (6110.00–6701.00) | 6270.00 (6095.00–6510.00) | 6170.00 (5992.50–6455.00) | 5990.00 (5100.00–6182.50) | 6260.00 (6200.00–6580.00) | 6240.00 (6180.00–6570.00) | 6230.00 (6100.00–6410.00) | 6245.00 (6050.00–6400.00) | **<0.001** | **0.011** | 0.758 | 0.631 | 0.304 | **0.019** |
| **Movement time, left lower diagonal (ms)** | 5795.00 (5617.50–6010.00) | 5735.00 (5597.50–5975.00) | 5670.00 (5550.00–5832.50) | 5602.50 (4960.00–5838.75) | 5830.00 (5710.00–6100.00) | 5750.00 (5680.00–6000.00) | 5700.00 (5600.00–5860.00) | 5735.00 (5430.00–5900.00) | **<0.001** | **0.012** | 0.493 | 0.583 | 0.391 | 0.239 |
| **Movement time, right lower diagonal (ms)** | 4715.00 (4550.00–4965.00) | 4645.00 (4538.75–4875.00) | 4590.00 (4522.50–4812.50) | 4542.50 (4337.50–4733.75) | 4710.00 (4610.00–5100.00) | 4650.00 (4580.00–4950.00) | 4610.00 (4500.00–4840.00) | 4615.00 (4515.00–4765.00) | **<0.001** | **0.006** | 0.664 | 0.656 | 0.715 | 0.268 |
| **OMEGO — Balance & Force Assessment** | | | | | | | | | | | | | | |
| **Weight distribution, prosthetic side (%)** | 34.00 (33.75–35.00) | 35.00 (34.00–41.25) | 35.00 (34.00–46.00) | 35.00 (34.00–49.00) | 35.00 (34.00–35.00) | 35.00 (34.00–39.00) | 35.00 (34.00–45.00) | 35.00 (34.00–49.00) | **0.011** | **0.018** | 0.600 | 0.587 | 0.540 | 0.603 |
| **Weight distribution, healthy side (%)** | 66.00 (65.00–66.25) | 65.00 (58.75–66.00) | 65.00 (54.00–66.00) | 65.00 (51.00–66.00) | 65.00 (65.00–66.00) | 65.00 (61.00–66.00) | 65.00 (55.00–66.00) | 65.00 (51.00–66.00) | **0.011** | **0.018** | 0.600 | 0.587 | 0.540 | 0.603 |
| **Proprioception difference, mean (°)** | 72.00 (68.00–75.50) | 56.00 (50.50–70.25) | 43.00 (37.50–71.25) | 35.50 (30.75–72.25) | 70.00 (66.00–73.00) | 58.00 (48.00–70.00) | 45.00 (36.00–71.00) | 37.00 (29.00–72.00) | **0.004** | **0.004** | 0.314 | 0.954 | 0.936 | 0.954 |
| **Force sensation, healthy limb (kg)** | 71.50 (70.10–76.15) | 71.05 (69.45–73.07) | 70.05 (67.28–71.20) | 69.15 (64.70–70.50) | 71.00 (69.20–73.20) | 70.40 (69.00–72.60) | 69.70 (66.90–71.40) | 69.20 (64.60–70.40) | **0.002** | **0.001** | 0.398 | 0.515 | 0.828 | 0.873 |
| **Force sensation, affected limb (kg)** | 38.30 (34.20–40.15) | 41.25 (39.95–46.50) | 47.60 (40.58–52.75) | 50.95 (40.67–56.33) | 39.30 (35.80–40.00) | 41.10 (39.70–46.90) | 45.10 (40.00–50.80) | 47.10 (40.10–53.60) | **0.002** | **0.001** | 0.873 | 0.784 | 0.417 | 0.373 |
| **STRIDE ONE System** | | | | | | | | | | | | | | |
| **Smoothness Index (a.u.)** | 16.35 (15.38–19.05) | 15.10 (12.45–16.10) | 14.70 (11.88–15.53) | 13.65 (10.17–14.93) | 16.90 (15.40–20.20) | 15.90 (14.70–16.40) | 14.60 (13.30–15.70) | 14.80 (12.10–16.30) | **0.001** | **0.006** | 0.486 | 0.309 | 0.537 | 0.197 |
| **Symmetry Index (%)** | 58.10 (53.80–61.40) | 60.05 (55.93–67.55) | 81.50 (57.82–86.92) | 85.70 (56.55–92.17) | 60.80 (54.10–66.80) | 61.90 (57.40–76.90) | 80.70 (57.70–85.90) | 84.80 (56.50–90.80) | **0.003** | **0.004** | 0.268 | 0.673 | 0.656 | 0.615 |
| **Roll-off quality, healthy limb (%)** | 86.00 (81.25–89.00) | 92.00 (89.25–96.25) | 93.50 (88.25–97.25) | 95.00 (89.25–97.25) | 87.00 (86.00–90.00) | 88.00 (85.00–90.00) | 86.00 (83.00–89.00) | 87.00 (84.00–90.00) | **<0.001** | 0.055 | 0.138 | **0.022** | **0.003** | **0.008** |
| **Roll-off quality, affected limb (%)** | 48.50 (42.75–59.00) | 81.50 (49.75–88.25) | 86.00 (48.75–92.25) | 88.50 (49.75–91.00) | 49.00 (47.00–59.00) | 50.00 (48.00–68.00) | 49.00 (47.00–74.00) | 50.00 (47.00–82.00) | **<0.001** | **0.003** | 0.606 | 0.066 | **0.041** | **0.042** |
| **Stance ratio, healthy limb (%)** | 54.00 (48.75–58.50) | 59.50 (55.75–62.25) | 57.50 (55.50–60.25) | 58.00 (55.00–61.25) | 57.00 (54.00–62.00) | 58.00 (55.00–62.00) | 57.00 (54.00–63.00) | 57.00 (55.00–63.00) | 0.087 | **0.014** | 0.090 | 0.622 | 0.909 | 1.000 |
| **Stance ratio, affected limb (%)** | 77.50 (74.50–81.25) | 74.50 (71.00–77.00) | 72.00 (70.75–74.50) | 73.00 (71.00–75.25) | 79.00 (77.00–83.00) | 77.00 (76.00–79.00) | 78.00 (77.00–80.00) | 77.00 (76.00–79.00) | **<0.001** | **0.043** | 0.119 | **0.011** | **<0.001** | **0.002** |
| **Step duration, healthy limb (s)** | 2.09 (1.94–2.28) | 1.90 (1.67–2.10) | 1.21 (1.10–2.04) | 1.21 (1.12–2.06) | 2.08 (1.98–2.12) | 2.08 (1.90–2.13) | 2.08 (2.00–2.12) | 2.10 (2.01–2.16) | **0.001** | 0.058 | 0.423 | 0.061 | **0.001** | **0.001** |
| **Step duration, affected limb (s)** | 2.50 (2.35–2.76) | 2.13 (1.86–2.38) | 1.70 (1.49–2.37) | 1.42 (1.35–2.38) | 2.38 (2.28–2.41) | 2.37 (2.25–2.40) | 2.39 (2.29–2.45) | 2.38 (2.28–2.44) | **<0.001** | 0.678 | 0.076 | 0.059 | **0.011** | **0.006** |
| **BALO Platform** | | | | | | | | | | | | | | |
| **Anterior-posterior stability index (a.u.)** | 1240.00 (1223.25–1261.25) | 2405.00 (1265.00–2520.00) | 4175.00 (1254.75–5060.00) | 5650.00 (1275.00–7480.00) | 1245.00 (1228.00–1265.00) | 1260.00 (1240.00–2450.00) | 1255.00 (1180.00–4980.00) | 1270.00 (980.00–7350.00) | **<0.001** | 0.093 | 0.656 | 0.120 | 0.110 | 0.102 |
| **Dynamic mediolateral stability index (a.u.)** | 1250.00 (1235.00–1266.75) | 1917.50 (1246.50–2698.75) | 2812.50 (1255.50–5295.00) | 3869.00 (1255.00–7710.00) | 1245.00 (1238.00–1260.00) | 1262.00 (1215.00–2665.00) | 1255.00 (1160.00–5210.00) | 1270.00 (945.00–7590.00) | **<0.001** | 0.092 | 0.775 | 0.123 | 0.178 | 0.213 |
| **Lateral stability index (a.u.)** | 1252.50 (1234.25–1270.00) | 2550.00 (1254.25–2868.75) | 4385.00 (1251.00–5515.00) | 6620.00 (1263.75–7880.00) | 1255.00 (1230.00–1268.00) | 1260.00 (1235.00–2835.00) | 1258.00 (1190.00–5480.00) | 1270.00 (1005.00–7850.00) | **<0.001** | 0.092 | 0.792 | 0.221 | 0.185 | 0.181 |
| **Functional Tests & Clinical Scales** | | | | | | | | | | | | | | |
| **Functional Reach Test (cm)** | 11.50 (9.00–13.25) | 17.00 (14.50–19.25) | 18.50 (13.75–22.00) | 20.50 (14.50–24.25) | 11.00 (9.00–12.00) | 11.00 (10.00–13.00) | 12.00 (9.00–13.00) | 11.00 (10.00–13.00) | **<0.001** | 0.240 | 0.303 | **<0.001** | **<0.001** | **0.002** |
| **Berg Balance Scale (pts)** | 27.00 (24.00–30.50) | 34.00 (31.75–37.00) | 43.00 (37.25–48.25) | 44.50 (37.25–49.25) | 34.00 (32.00–37.00) | 35.00 (34.00–36.00) | 35.00 (34.00–38.00) | 35.00 (33.00–38.00) | **<0.001** | 0.173 | **0.003** | 0.232 | **0.010** | **0.003** |
| **Timed Up and Go (s)** | 31.50 (28.50–36.25) | 22.50 (19.75–25.50) | 13.50 (10.75–25.75) | 13.50 (10.75–24.75) | 30.00 (29.00–38.00) | 30.00 (28.00–31.00) | 31.00 (28.00–32.00) | 30.00 (28.00–31.00) | **<0.001** | 0.275 | 0.827 | **0.001** | **<0.001** | **<0.001** |
| **Amputee Mobility Predictor (pts)** | 22.50 (20.75–25.00) | 31.50 (24.50–33.25) | 37.00 (24.75–39.25) | 43.50 (24.75–47.25) | 23.00 (23.00–25.00) | 24.00 (23.00–27.00) | 24.00 (23.00–25.00) | 24.00 (22.00–25.00) | **<0.001** | 0.188 | 0.183 | **0.028** | **0.012** | **0.008** |
| **Four Square Step Test (s)** | 22.50 (18.00–25.00) | 16.00 (14.00–17.00) | 13.50 (12.00–17.25) | 12.00 (10.00–16.25) | 23.00 (20.00–24.00) | 21.00 (16.00–22.00) | 22.00 (17.00–23.00) | 21.00 (16.00–22.00) | **<0.001** | **0.044** | 1.000 | **0.006** | **<0.001** | **<0.001** |
| **10-Meter Walk Test (m/s)** | 0.41 (0.35–0.47) | 0.46 (0.42–0.52) | 0.83 (0.48–0.89) | 0.96 (0.47–1.11) | 0.41 (0.40–0.45) | 0.42 (0.41–0.52) | 0.43 (0.42–0.62) | 0.42 (0.41–0.78) | **<0.001** | **<0.001** | 0.558 | 0.456 | **0.036** | **0.018** |
| **2-Minute Walk Test (m)** | 52.00 (43.75–62.75) | 65.00 (59.00–72.75) | 84.50 (66.00–98.25) | 122.00 (65.75–135.25) | 56.00 (52.00–61.00) | 57.00 (55.00–64.00) | 57.00 (54.00–69.00) | 56.00 (53.00–69.00) | **<0.001** | **0.021** | 0.182 | 0.050 | **0.015** | **0.011** |
| **6-Minute Walk Test (m)** | 166.50 (143.00–206.25) | 213.50 (177.50–242.00) | 281.50 (201.00–316.75) | 399.50 (201.25–458.25) | 165.00 (149.00–198.00) | 170.00 (162.00–190.00) | 168.00 (160.00–212.00) | 166.00 (144.00–211.00) | **<0.001** | **0.021** | 0.756 | **0.017** | **0.003** | **0.002** |
| **Activities-specific Balance Confidence Scale (%)** | 42.00 (35.25–50.50) | 49.00 (42.25–56.00) | 56.00 (47.25–63.75) | 68.00 (52.75–78.50) | 44.00 (42.00–50.00) | 45.00 (43.00–51.00) | 42.00 (40.00–55.00) | 43.00 (37.00–56.00) | **<0.001** | **0.018** | 0.401 | 0.654 | **0.040** | **0.009** |
| **Visual Analog Scale — pain (pts)** | 3.00 (2.00–3.00) | 2.00 (2.00–2.25) | 1.50 (1.00–2.00) | 1.00 (1.00–2.00) | 5.00 (4.00–6.00) | 4.00 (3.00–4.00) | 3.00 (2.00–4.00) | 3.00 (2.00–4.00) | **<0.001** | **<0.001** | **<0.001** | **<0.001** | **<0.001** | **<0.001** |
| **PTSD Checklist (PCL-5) (pts)** | 29.50 (28.00–31.25) | — | — | 21.00 (18.50–23.25) | 66.00 (61.00–69.00) | — | — | 55.00 (48.00–58.00) | **<0.001** | **<0.001** | **<0.001** | — | — | **<0.001** |
| **Patient Health Questionnaire (PHQ-9) (pts)** | 13.50 (11.75–14.00) | — | — | 10.00 (8.00–12.00) | 18.00 (16.00–19.00) | — | — | 13.00 (11.00–15.00) | **<0.001** | **<0.001** | **<0.001** | — | — | **0.004** |

**Supplementary Table S4. Stratification by PHQ-9 Level (Depressive Severity)**

Group 0: PHQ-9 <15 (mild depression) (n=22) vs Group 1: PHQ-9 ≥15 (mod–severe) (n=23). Within-group p = Wilcoxon signed-rank (D0→D120). Between-group p = Mann–Whitney U at each timepoint. Bold terracotta = p < 0.05.

| **Variable** | **Group 0: PHQ-9 <15 (mild depression) (n=22)** | | | | **Group 1: PHQ-9 ≥15 (mod–severe) (n=23)** | | | | **Within-group p (D0→D120)** | | **Between-group p (Mann–Whitney U)** | | | |
| --- | --- | --- | --- | --- | --- | --- | --- | --- | --- | --- | --- | --- | --- | --- |
|  | **D0** | **D14** | **D30** | **D120** | **D0** | **D14** | **D30** | **D120** | **p G0** | **p G1** | **D0** | **D14** | **D30** | **D120** |
| **Stabiloplatforma Alfa — Romberg Test** | | | | | | | | | | | | | | |
| **COP X-axis displacement, eyes open (cm)** | 2.33 (2.25–2.47) | 2.24 (1.90–2.37) | 2.18 (1.69–2.30) | 2.03 (1.53–2.25) | 2.18 (2.12–2.23) | 2.15 (2.10–2.24) | 2.17 (2.08–2.24) | 2.18 (2.09–2.26) | **<0.001** | **0.036** | **0.006** | 0.256 | 0.683 | 0.169 |
| **COP X-axis displacement, eyes closed (cm)** | 2.76 (2.72–2.84) | 2.62 (2.44–2.75) | 2.42 (2.08–2.71) | 2.36 (1.98–2.72) | 2.72 (2.69–2.81) | 2.71 (2.69–2.79) | 2.71 (2.67–2.79) | 2.72 (2.68–2.80) | **<0.001** | **0.036** | 0.169 | **0.040** | **0.012** | **0.002** |
| **COP Y-axis displacement, eyes open (cm)** | 2.84 (2.77–2.88) | 2.72 (2.36–2.85) | 2.46 (2.12–2.82) | 2.38 (1.97–2.83) | 2.82 (2.76–2.91) | 2.81 (2.76–2.88) | 2.79 (2.74–2.88) | 2.80 (2.75–2.89) | **<0.001** | **0.027** | 0.741 | 0.059 | **0.021** | **0.006** |
| **COP Y-axis displacement, eyes closed (cm)** | 3.69 (3.62–3.76) | 3.64 (3.27–3.67) | 3.35 (3.07–3.63) | 3.19 (2.76–3.60) | 3.67 (3.61–3.76) | 3.69 (3.60–3.76) | 3.68 (3.58–3.76) | 3.69 (3.59–3.77) | **<0.001** | **0.035** | 0.699 | **0.035** | **<0.001** | **<0.001** |
| **Mean velocity X-axis, eyes open (cm/s)** | 1.46 (1.35–1.54) | 1.29 (1.09–1.43) | 1.23 (0.79–1.31) | 0.93 (0.58–1.29) | 1.33 (1.18–1.43) | 1.27 (1.06–1.40) | 1.25 (1.06–1.37) | 1.27 (0.96–1.39) | **<0.001** | **0.009** | **0.013** | 0.578 | 0.433 | 0.114 |
| **Mean velocity X-axis, eyes closed (cm/s)** | 3.20 (3.04–4.31) | 3.13 (2.91–3.37) | 2.94 (2.80–3.10) | 2.16 (1.98–2.88) | 3.18 (2.88–3.92) | 3.12 (2.90–3.62) | 3.09 (2.81–3.27) | 2.99 (2.46–3.37) | **<0.001** | **0.009** | 0.192 | 0.847 | 0.173 | **0.015** |
| **Mean velocity Y-axis, eyes open (cm/s)** | 1.43 (1.35–1.55) | 1.27 (1.11–1.42) | 1.23 (0.93–1.29) | 1.11 (0.84–1.29) | 1.34 (1.21–1.47) | 1.32 (1.07–1.44) | 1.34 (1.02–1.46) | 1.33 (1.16–1.50) | **<0.001** | **0.023** | 0.084 | 0.776 | 0.251 | **0.043** |
| **Mean velocity Y-axis, eyes closed (cm/s)** | 3.35 (2.81–4.09) | 2.73 (2.45–3.25) | 2.70 (2.26–3.11) | 2.27 (1.63–2.85) | 3.45 (2.92–3.74) | 3.22 (2.31–3.61) | 3.30 (2.33–3.60) | 3.27 (1.95–3.70) | **<0.001** | **0.016** | 0.578 | 0.143 | 0.063 | **0.015** |
| **Area of oscillation, eyes open (cm²)** | 15.05 (14.60–15.78) | 14.80 (13.60–15.32) | 14.30 (11.28–15.20) | 14.45 (10.53–15.30) | 15.10 (14.60–15.65) | 14.70 (12.30–15.75) | 14.60 (10.00–16.30) | 14.60 (9.30–15.95) | **0.001** | **0.018** | 0.750 | 0.699 | 0.460 | 0.650 |
| **Area of oscillation, eyes closed (cm²)** | 23.95 (22.80–24.73) | 22.30 (19.73–23.73) | 22.15 (16.32–23.77) | 22.30 (13.35–23.95) | 23.50 (22.85–24.30) | 22.80 (18.40–24.65) | 22.60 (15.60–25.65) | 22.80 (13.10–24.30) | **<0.001** | **0.007** | 0.633 | 0.547 | 0.539 | 0.683 |
| **COP path length, eyes open (cm)** | 36.50 (35.15–39.05) | 36.30 (34.65–38.88) | 34.75 (31.77–36.40) | 34.80 (27.93–36.60) | 36.60 (35.70–40.65) | 36.80 (34.80–39.05) | 35.10 (28.20–40.35) | 35.30 (25.90–38.35) | **0.001** | **0.017** | 0.725 | 0.733 | 0.467 | 0.540 |
| **COP path length, eyes closed (cm)** | 89.90 (87.38–91.85) | 86.90 (75.50–90.22) | 86.50 (63.42–89.92) | 86.90 (40.10–89.35) | 88.90 (87.00–90.85) | 88.00 (74.60–93.50) | 87.70 (64.45–97.80) | 88.10 (43.45–103.65) | **<0.001** | 0.063 | 0.323 | 0.532 | 0.440 | 0.281 |
| **Stabiloplatforma Alfa — Unterberger Test** | | | | | | | | | | | | | | |
| **Angle deviation per step (°)** | 6.65 (6.33–6.90) | 6.55 (5.90–6.90) | 6.30 (4.55–6.70) | 6.35 (3.96–6.80) | 6.60 (6.50–7.00) | 6.70 (6.20–7.00) | 6.40 (5.00–7.25) | 6.50 (4.35–7.65) | **0.007** | **0.027** | 0.657 | 0.439 | 0.388 | 0.363 |
| **Movement amplitude (°)** | 15.40 (14.83–16.18) | 14.85 (13.57–15.78) | 14.75 (10.98–15.62) | 14.80 (7.92–15.73) | 15.50 (14.70–16.15) | 15.30 (13.95–16.10) | 15.00 (11.40–17.25) | 15.10 (8.55–18.25) | **0.001** | 0.106 | 0.973 | 0.340 | 0.352 | 0.352 |
| **Stabiloplatforma Alfa — Movement Time Test** | | | | | | | | | | | | | | |
| **Movement time, left upper diagonal (ms)** | 4240.00 (4122.50–4437.50) | 4190.00 (4088.75–4420.00) | 4175.00 (4057.50–4407.50) | 4130.00 (3657.50–4352.50) | 4290.00 (4210.00–4513.50) | 4230.00 (4180.00–4400.00) | 4190.00 (4075.00–4370.00) | 4200.00 (3875.00–4377.50) | **<0.001** | **0.013** | 0.510 | 0.633 | 0.901 | 0.617 |
| **Movement time, right upper diagonal (ms)** | 6345.00 (6165.00–6619.75) | 6245.00 (6122.50–6517.50) | 6185.00 (6047.50–6485.00) | 6095.00 (5322.50–6348.75) | 6340.00 (6215.00–6742.50) | 6250.00 (6180.00–6535.00) | 6180.00 (6075.00–6405.00) | 6185.00 (5840.00–6400.00) | **<0.001** | **0.004** | 0.683 | 0.794 | 0.964 | 0.401 |
| **Movement time, left lower diagonal (ms)** | 5810.00 (5665.00–6070.00) | 5725.00 (5635.00–6025.00) | 5695.00 (5586.25–5857.50) | 5630.00 (5407.50–5868.75) | 5800.00 (5715.00–6040.00) | 5750.00 (5685.00–5985.00) | 5680.00 (5575.00–5845.00) | 5735.00 (5415.00–5862.50) | **<0.001** | **0.038** | 0.570 | 0.625 | 0.883 | 0.586 |
| **Movement time, right lower diagonal (ms)** | 4705.00 (4582.50–5085.00) | 4635.00 (4547.50–4965.00) | 4595.00 (4530.00–4837.50) | 4592.50 (4453.50–4771.25) | 4730.00 (4615.00–5000.00) | 4650.00 (4585.00–4900.00) | 4610.00 (4500.00–4820.00) | 4596.00 (4370.50–4712.50) | **<0.001** | **0.002** | 0.555 | 0.716 | 0.937 | 0.856 |
| **OMEGO — Balance & Force Assessment** | | | | | | | | | | | | | | |
| **Weight distribution, prosthetic side (%)** | 34.00 (34.00–35.00) | 35.00 (34.00–39.75) | 35.00 (34.00–46.00) | 35.00 (34.25–49.00) | 35.00 (33.50–35.00) | 35.00 (33.00–40.00) | 35.00 (32.50–45.00) | 35.00 (30.50–49.00) | **0.007** | **0.026** | 0.840 | 0.498 | 0.318 | 0.346 |
| **Weight distribution, healthy side (%)** | 66.00 (65.00–66.00) | 65.00 (60.25–66.00) | 65.00 (54.00–66.00) | 65.00 (51.00–65.75) | 65.00 (65.00–66.50) | 65.00 (60.00–67.00) | 65.00 (55.00–67.50) | 65.00 (51.00–69.50) | **0.007** | **0.026** | 0.840 | 0.498 | 0.318 | 0.346 |
| **Proprioception difference, mean (°)** | 72.00 (68.00–74.00) | 54.50 (48.25–69.50) | 41.50 (36.00–70.25) | 34.50 (29.25–71.25) | 70.00 (66.50–75.00) | 58.00 (51.00–70.50) | 46.00 (38.00–71.50) | 38.00 (30.50–72.50) | **0.001** | **0.011** | 0.633 | 0.666 | 0.601 | 0.609 |
| **Force sensation, healthy limb (kg)** | 71.50 (69.58–74.30) | 70.85 (69.08–72.25) | 69.40 (66.22–71.10) | 68.80 (63.77–70.85) | 70.80 (69.60–75.35) | 70.50 (69.40–73.45) | 70.10 (68.40–71.60) | 69.60 (66.35–70.30) | **0.001** | **0.002** | 0.919 | 0.865 | 0.555 | 0.666 |
| **Force sensation, affected limb (kg)** | 39.10 (35.50–40.62) | 42.25 (40.38–47.55) | 47.15 (40.75–51.50) | 50.55 (40.85–55.12) | 37.10 (33.00–39.95) | 40.40 (39.65–44.35) | 45.10 (39.95–52.85) | 47.10 (40.05–56.90) | **0.001** | **0.002** | 0.352 | 0.188 | 0.691 | 0.641 |
| **STRIDE ONE System** | | | | | | | | | | | | | | |
| **Smoothness Index (a.u.)** | 16.40 (15.22–20.33) | 15.45 (14.55–16.38) | 14.55 (12.53–15.78) | 13.35 (10.80–14.95) | 16.80 (15.80–19.30) | 15.50 (14.15–16.35) | 14.80 (13.05–15.70) | 14.90 (11.50–16.20) | **<0.001** | **0.007** | 0.742 | 0.892 | 0.699 | 0.212 |
| **Symmetry Index (%)** | 58.60 (53.60–65.90) | 60.05 (54.45–74.90) | 81.50 (58.85–86.88) | 85.70 (57.62–91.97) | 60.20 (56.55–63.95) | 61.90 (57.15–73.55) | 80.70 (57.05–85.05) | 84.80 (55.85–89.95) | **<0.001** | **0.012** | 0.454 | 0.794 | 0.625 | 0.601 |
| **Roll-off quality, healthy limb (%)** | 86.00 (84.25–89.00) | 91.50 (87.25–96.75) | 92.50 (86.25–96.00) | 95.00 (87.25–97.75) | 87.00 (83.50–90.00) | 88.00 (84.50–90.00) | 86.00 (82.50–89.50) | 87.00 (83.50–90.50) | **<0.001** | **0.022** | 0.715 | **0.017** | **0.014** | **0.012** |
| **Roll-off quality, affected limb (%)** | 49.00 (46.50–61.75) | 81.50 (49.25–88.00) | 85.50 (48.25–92.75) | 87.50 (49.25–91.00) | 49.00 (45.50–53.00) | 50.00 (47.50–66.50) | 49.00 (46.50–72.50) | 50.00 (46.50–80.50) | **<0.001** | **0.002** | 0.459 | **0.033** | **0.018** | **0.024** |
| **Stance ratio, healthy limb (%)** | 55.00 (52.25–61.50) | 59.00 (56.00–62.75) | 58.50 (56.00–61.00) | 58.00 (55.00–61.75) | 55.00 (52.50–58.50) | 58.00 (55.00–62.00) | 56.00 (54.00–62.50) | 57.00 (54.50–62.00) | 0.136 | **0.006** | 0.927 | 0.368 | 0.459 | 0.600 |
| **Stance ratio, affected limb (%)** | 79.00 (76.25–81.75) | 75.50 (71.25–77.75) | 73.00 (70.25–77.00) | 74.00 (71.00–76.00) | 78.00 (77.00–82.00) | 77.00 (75.50–79.00) | 78.00 (74.50–80.00) | 77.00 (74.50–79.00) | **0.002** | **0.035** | 0.724 | 0.089 | **0.010** | **0.020** |
| **Step duration, healthy limb (s)** | 2.10 (1.93–2.23) | 1.94 (1.71–2.11) | 1.25 (1.16–2.08) | 1.21 (1.11–2.10) | 2.08 (1.99–2.12) | 2.06 (1.88–2.12) | 2.08 (1.85–2.12) | 2.09 (1.86–2.13) | **0.002** | 0.081 | 0.433 | 0.237 | **0.023** | **0.012** |
| **Step duration, affected limb (s)** | 2.41 (2.32–2.70) | 2.24 (1.91–2.39) | 1.71 (1.51–2.38) | 1.42 (1.31–2.38) | 2.38 (2.30–2.44) | 2.37 (2.18–2.41) | 2.39 (2.16–2.45) | 2.38 (2.15–2.44) | **0.001** | 0.594 | 0.270 | 0.086 | **0.018** | **0.010** |
| **BALO Platform** | | | | | | | | | | | | | | |
| **Anterior-posterior stability index (a.u.)** | 1242.50 (1230.00–1263.75) | 2405.00 (1264.00–2540.00) | 3675.00 (1258.00–5040.00) | 5650.00 (1276.25–7465.00) | 1240.00 (1222.50–1265.00) | 1260.00 (1229.00–2417.50) | 1242.00 (1197.50–4987.50) | 1260.00 (1107.50–7385.00) | **<0.001** | 0.055 | 0.794 | **0.028** | 0.192 | 0.140 |
| **Dynamic mediolateral stability index (a.u.)** | 1245.00 (1229.75–1267.25) | 1917.50 (1240.50–2716.25) | 2811.00 (1231.25–5273.75) | 3869.00 (1250.00–7702.50) | 1250.00 (1239.00–1260.00) | 1268.00 (1227.50–2672.50) | 1261.00 (1196.50–5220.00) | 1275.00 (1110.00–7635.00) | **0.003** | 0.055 | 0.794 | 0.370 | 0.570 | 0.586 |
| **Lateral stability index (a.u.)** | 1260.00 (1235.00–1268.00) | 1812.50 (1243.75–2886.25) | 2761.00 (1249.00–5506.25) | 3807.50 (1252.25–7872.50) | 1248.00 (1229.00–1270.00) | 1260.00 (1239.00–2847.50) | 1258.00 (1230.00–5497.50) | 1270.00 (1233.00–7865.00) | **0.002** | **0.018** | 0.811 | 0.510 | 0.510 | 0.467 |
| **Functional Tests & Clinical Scales** | | | | | | | | | | | | | | |
| **Functional Reach Test (cm)** | 11.00 (9.00–12.00) | 16.50 (12.00–19.75) | 16.50 (12.25–20.75) | 20.50 (12.00–24.75) | 11.00 (9.50–12.00) | 11.00 (10.00–14.00) | 12.00 (10.00–14.00) | 11.00 (10.00–14.00) | **<0.001** | 0.104 | 0.827 | **0.006** | **0.012** | **0.010** |
| **Berg Balance Scale (pts)** | 27.50 (24.00–33.50) | 34.50 (33.00–36.75) | 43.00 (35.00–48.75) | 43.50 (34.25–47.75) | 34.00 (31.00–36.50) | 35.00 (33.00–36.00) | 35.00 (34.00–38.00) | 35.00 (32.50–38.50) | **<0.001** | 0.083 | **0.011** | 0.740 | **0.014** | **0.031** |
| **Timed Up and Go (s)** | 31.00 (29.00–36.75) | 23.50 (20.25–29.00) | 14.50 (11.25–30.00) | 14.50 (11.25–29.00) | 30.00 (28.50–36.50) | 30.00 (25.50–31.00) | 31.00 (26.00–32.00) | 30.00 (25.50–31.00) | **<0.001** | 0.129 | 0.607 | **0.027** | **0.025** | **0.023** |
| **Amputee Mobility Predictor (pts)** | 23.00 (22.00–25.00) | 30.00 (24.00–33.00) | 36.00 (23.25–38.75) | 43.00 (24.00–46.50) | 23.00 (22.00–25.00) | 24.00 (23.00–27.00) | 24.00 (23.00–25.00) | 24.00 (22.50–25.50) | **<0.001** | 0.140 | 0.963 | 0.060 | 0.081 | 0.056 |
| **Four Square Step Test (s)** | 22.50 (19.25–25.00) | 16.00 (14.00–20.25) | 14.50 (12.00–21.00) | 12.50 (11.00–20.00) | 23.00 (18.00–24.00) | 21.00 (16.50–22.00) | 22.00 (16.00–23.00) | 21.00 (15.00–22.00) | **<0.001** | **0.015** | 0.819 | **0.022** | **0.008** | **0.010** |
| **10-Meter Walk Test (m/s)** | 0.41 (0.37–0.48) | 0.48 (0.42–0.55) | 0.82 (0.45–0.90) | 0.94 (0.44–1.07) | 0.41 (0.37–0.45) | 0.42 (0.41–0.49) | 0.43 (0.42–0.62) | 0.42 (0.41–0.78) | **<0.001** | **<0.001** | 0.411 | 0.073 | **0.023** | **0.033** |
| **2-Minute Walk Test (m)** | 53.50 (45.25–59.75) | 63.50 (57.00–71.50) | 82.50 (57.00–100.75) | 122.00 (56.00–135.75) | 58.00 (52.00–61.00) | 57.00 (55.00–66.50) | 57.00 (54.00–69.00) | 56.00 (53.00–70.00) | **<0.001** | **0.010** | 0.337 | 0.104 | **0.028** | **0.024** |
| **6-Minute Walk Test (m)** | 165.00 (145.25–208.75) | 208.50 (172.50–230.00) | 268.50 (168.00–306.25) | 394.50 (167.50–462.25) | 165.00 (146.00–198.00) | 170.00 (162.00–190.50) | 168.00 (155.00–212.00) | 166.00 (144.00–214.50) | **<0.001** | **0.010** | 0.648 | **0.027** | **0.014** | **0.007** |
| **Activities-specific Balance Confidence Scale (%)** | 42.00 (36.00–51.50) | 50.50 (43.00–56.00) | 55.00 (43.00–65.00) | 64.00 (43.00–75.75) | 44.00 (39.00–49.00) | 45.00 (41.50–50.50) | 42.00 (39.50–55.00) | 43.00 (37.00–56.00) | **<0.001** | **0.008** | 0.792 | 0.231 | **0.045** | **0.026** |
| **Visual Analog Scale — pain (pts)** | 3.00 (2.00–3.00) | 2.00 (2.00–2.00) | 1.50 (1.00–2.00) | 1.00 (1.00–2.00) | 5.00 (4.00–6.00) | 4.00 (3.00–5.00) | 3.00 (2.00–4.00) | 3.00 (2.00–4.00) | **<0.001** | **<0.001** | **<0.001** | **<0.001** | **<0.001** | **<0.001** |
| **PTSD Checklist (PCL-5) (pts)** | 30.00 (28.25–32.00) | — | — | 21.00 (19.25–27.75) | 66.00 (60.50–69.00) | — | — | 54.00 (40.00–57.50) | **<0.001** | **<0.001** | **<0.001** | — | — | **<0.001** |
| **Patient Health Questionnaire (PHQ-9) (pts)** | 13.50 (12.00–14.00) | — | — | 9.50 (8.00–10.75) | 18.00 (17.00–19.00) | — | — | 14.00 (12.00–15.00) | **<0.001** | **<0.001** | **<0.001** | — | — | **<0.001** |

**Supplementary Table S5. Sample Size and Statistical Power Analysis**

Retrospective power analysis based on observed Cohen's d for pre–post change scores. Power = Φ(|d|√n − zα/2), α = 0.05, two-tailed. Green = power ≥80%; red = power < 80%.

**5A. Within-Group Power (Pre–Post, n = 45)**

| **Outcome** | **Mean Δ** | **SD Δ** | **Cohen's d** | **Observed Power (n = 45)** | **n Required (80%)** | **n Required (90%)** | **Wilcoxon p (D0→D120)** |
| --- | --- | --- | --- | --- | --- | --- | --- |
| **BBS (pts)** | 8.42 | 11.96 | 0.704 | **99.7%** | 16 | 22 | **<0.001** |
| **TUG (s)** | -7.09 | 12.08 | -0.587 | **97.6%** | 23 | 31 | **<0.001** |
| **FRT (cm)** | 4.8 | 6.75 | 0.711 | **99.8%** | 16 | 21 | **<0.001** |
| **ABC (%)** | 11.31 | 17.07 | 0.663 | **99.4%** | 18 | 24 | **<0.001** |
| **6MWT (m)** | 95.69 | 136.75 | 0.7 | **99.7%** | 17 | 22 | **<0.001** |
| **VAS (pts)** | -1.64 | 1.33 | -1.233 | **100.0%** | 6 | 7 | **<0.001** |
| **PCL-5 (pts)** | -11.38 | 6.11 | -1.861 | **100.0%** | 3 | 4 | **<0.001** |
| **PHQ-9 (pts)** | -3.89 | 2.35 | -1.657 | **100.0%** | 3 | 4 | **<0.001** |
| **AMPPRO (pts)** | 8.22 | 11.52 | 0.713 | **99.8%** | 16 | 21 | **<0.001** |
| **2MWT (m)** | 30.64 | 42.75 | 0.717 | **99.8%** | 16 | 21 | **<0.001** |
| **FSST (s)** | -3.93 | 7.4 | -0.532 | **94.6%** | 28 | 38 | **<0.001** |

**5B. Between-Group Power (PCL-5 Stratification)**

Between-group Cohen's d on change scores (ΔD0→D120). Harmonic-mean power. Mann–Whitney U p-values.

| **Outcome** | **n G0 (Low)** | **n G1 (High)** | **Cohen's d** | **Power** | **Mann–Whitney p** |
| --- | --- | --- | --- | --- | --- |
| **ΔBBS** | 20 | 25 | 1.174 | **100.0%** | **0.001** |
| **ΔTUG** | 20 | 25 | -1.312 | **100.0%** | **<0.001** |
| **Δ6MWT** | 20 | 25 | 1.22 | **100.0%** | **<0.001** |
| **ΔFRT** | 20 | 25 | 1.006 | **99.7%** | **<0.001** |
| **ΔABC** | 20 | 25 | 1.202 | **100.0%** | **<0.001** |
| **ΔAMPPRO** | 20 | 25 | 1.211 | **100.0%** | **0.001** |

**Supplementary Table S6. Exploratory Predictive Analyses: ROC and Random Forest**

All results in this table are exploratory. Analyses were not pre-registered, were not adjusted for multiple comparisons, and were not validated in an independent sample. Interpret with caution.

| **6A. ROC Analysis — Composite Responder Prediction** | | | | | |
| --- | --- | --- | --- | --- | --- |
| **Predictor** | **AUC** | **95% CI (bootstrap)** | **n resp. / n total** | **Responder definition** | **Note** |
| **PCL-5** | 0.798 | 0.661 – 0.921 | 17 / 45 | ΔBBS ≥ 5 pts AND ΔTUG ≤ −5 s at Day 120 | Exploratory; not validated in independent sample |
| **PHQ-9** | 0.804 | 0.640 – 0.925 | 17 / 45 | ΔBBS ≥ 5 pts AND ΔTUG ≤ −5 s at Day 120 | Exploratory; not validated in independent sample |
| **PCL-5 + PHQ-9 + VAS (composite)** | 0.809 | 0.667 – 0.919 | 17 / 45 | ΔBBS ≥ 5 pts AND ΔTUG ≤ −5 s at Day 120 | Exploratory; not validated in independent sample |

| **6B. Random Forest Feature Importance — Psychiatric Predictors (Exploratory)** | | | | |
| --- | --- | --- | --- | --- |
| **Outcome** | **Predictor** | **Importance (mean)** | **Bootstrap 95% CI** | **Note** |
| **ΔBBS** | | | | |
| **ΔBBS** | PCL-5 | 0.569 | 0.275 – 0.807 | Exploratory; 500 trees, psych predictors only |
| **ΔBBS** | PHQ-9 | 0.323 | 0.124 – 0.619 | Exploratory; 500 trees, psych predictors only |
| **ΔBBS** | VAS | 0.107 | 0.038 – 0.186 | Exploratory; 500 trees, psych predictors only |
| **ΔTUG** | | | | |
| **ΔTUG** | PCL-5 | 0.590 | 0.312 – 0.790 | Exploratory; 500 trees, psych predictors only |
| **ΔTUG** | PHQ-9 | 0.299 | 0.114 – 0.580 | Exploratory; 500 trees, psych predictors only |
| **ΔTUG** | VAS | 0.111 | 0.045 – 0.211 | Exploratory; 500 trees, psych predictors only |
| **Δ6MWT** | | | | |
| **Δ6MWT** | PCL-5 | 0.560 | 0.278 – 0.820 | Exploratory; 500 trees, psych predictors only |
| **Δ6MWT** | PHQ-9 | 0.327 | 0.118 – 0.608 | Exploratory; 500 trees, psych predictors only |
| **Δ6MWT** | VAS | 0.113 | 0.031 – 0.204 | Exploratory; 500 trees, psych predictors only |

**Supplementary Table S7. Exploratory k-Means Cluster Characteristics**

k-Means clustering (k = 2, 20 restarts) applied to standardized baseline variables (PCL-5, PHQ-9, VAS, BBS, ABC, TUG). Exploratory; p-values not adjusted for multiple comparisons.

| **Variable** | **Cluster B: Resilient (n = 20) Median [IQR]** | **Cluster A: Burdened (n = 25) Median [IQR]** | **Mann–Whitney p** |
| --- | --- | --- | --- |
| **Baseline Characteristics** | | | |
| **PCL-5 (baseline)** | 30.0 [28.0–32.0] | 66.5 [60.8–69.2] | **< 0.001** |
| **PHQ-9 (baseline)** | 13.0 [12.0–14.0] | 18.0 [16.0–19.0] | **< 0.001** |
| **VAS (baseline)** | 3.0 [2.0–3.0] | 5.0 [4.0–6.0] | **< 0.001** |
| **BBS (baseline)** | 28.0 [24.0–32.0] | 34.0 [31.5–36.2] | **0.0164** |
| **TUG, s (baseline)** | 31.0 [29.0–37.0] | 30.5 [29.0–35.8] | 0.7924 |
| **ABC (baseline)** | 42.0 [36.0–50.0] | 44.0 [42.0–50.0] | 0.3129 |
| **Rehabilitation Change Scores (Day 120 – Day 0)** | | | |
| **ΔBBS** | 16.0 [1.0–24.0] | 0.5 [0.0–1.0] | **0.0230** |
| **ΔTUG, s** | -17.0 [-24.0–-1.0] | 0.0 [-1.0–0.0] | **0.0094** |
| **ΔFRT, cm** | 9.0 [1.0–12.0] | 0.0 [0.0–1.0] | **0.0100** |
| **ΔABC, %** | 29.0 [1.0–33.0] | 1.0 [1.0–1.0] | **0.0230** |
| **Δ6MWT, m** | 245.0 [5.0–270.0] | 1.0 [1.0–8.0] | **0.0131** |
| **ΔPCL-5** | -8.0 [-11.0–-5.0] | -12.0 [-13.0–-11.0] | **< 0.001** |
| **ΔPHQ-9** | -3.0 [-5.0–-2.0] | -5.0 [-5.0–-3.8] | 0.1306 |
| Exploratory analysis. Clustering was not pre-specified. p-values not adjusted for multiple comparisons. | | | |

**Supplementary Table S8. Exploratory Elastic Net Feature Selection**

Elastic Net regression (α = 0.5) with bootstrap selection frequency (100 iterations). Top 10 predictors per outcome listed by selection frequency. Exploratory; not validated in independent sample.

| **Outcome** | **Predictor (baseline)** | **Selection freq. (%)** | **Note** |
| --- | --- | --- | --- |
| **Outcome: ΔBBS** | | | |
| **ΔBBS** | BBS | **100%** | Bootstrap selection from 100 iterations (n=45, α=0.5). Exploratory only. |
| **ΔBBS** | PCL5 | **99%** | Bootstrap selection from 100 iterations (n=45, α=0.5). Exploratory only. |
| **ΔBBS** | PHQ9 | **96%** | Bootstrap selection from 100 iterations (n=45, α=0.5). Exploratory only. |
| **ΔBBS** | 10MWT | **87%** | Bootstrap selection from 100 iterations (n=45, α=0.5). Exploratory only. |
| **ΔBBS** | 6MWT | **82%** | Bootstrap selection from 100 iterations (n=45, α=0.5). Exploratory only. |
| **ΔBBS** | FS AFFECTED | **80%** | Bootstrap selection from 100 iterations (n=45, α=0.5). Exploratory only. |
| **ΔBBS** | RT PATH WEC | 76% | Bootstrap selection from 100 iterations (n=45, α=0.5). Exploratory only. |
| **ΔBBS** | MT LUD | 75% | Bootstrap selection from 100 iterations (n=45, α=0.5). Exploratory only. |
| **ΔBBS** | BALO DIM | 75% | Bootstrap selection from 100 iterations (n=45, α=0.5). Exploratory only. |
| **ΔBBS** | BAL PROS | 74% | Bootstrap selection from 100 iterations (n=45, α=0.5). Exploratory only. |
| **Outcome: ΔTUG** | | | |
| **ΔTUG** | BBS | **100%** | Bootstrap selection from 100 iterations (n=45, α=0.5). Exploratory only. |
| **ΔTUG** | PCL5 | **99%** | Bootstrap selection from 100 iterations (n=45, α=0.5). Exploratory only. |
| **ΔTUG** | PHQ9 | **95%** | Bootstrap selection from 100 iterations (n=45, α=0.5). Exploratory only. |
| **ΔTUG** | BALO DIM | **93%** | Bootstrap selection from 100 iterations (n=45, α=0.5). Exploratory only. |
| **ΔTUG** | ABC | **90%** | Bootstrap selection from 100 iterations (n=45, α=0.5). Exploratory only. |
| **ΔTUG** | 6MWT | **85%** | Bootstrap selection from 100 iterations (n=45, α=0.5). Exploratory only. |
| **ΔTUG** | SYMM IDX | **83%** | Bootstrap selection from 100 iterations (n=45, α=0.5). Exploratory only. |
| **ΔTUG** | STANCE AFF | **83%** | Bootstrap selection from 100 iterations (n=45, α=0.5). Exploratory only. |
| **ΔTUG** | FRT | **83%** | Bootstrap selection from 100 iterations (n=45, α=0.5). Exploratory only. |
| **ΔTUG** | RT PATH WEC | **81%** | Bootstrap selection from 100 iterations (n=45, α=0.5). Exploratory only. |
| **Outcome: Δ6MWT** | | | |
| **Δ6MWT** | BBS | **100%** | Bootstrap selection from 100 iterations (n=45, α=0.5). Exploratory only. |
| **Δ6MWT** | ABC | **100%** | Bootstrap selection from 100 iterations (n=45, α=0.5). Exploratory only. |
| **Δ6MWT** | PCL5 | **100%** | Bootstrap selection from 100 iterations (n=45, α=0.5). Exploratory only. |
| **Δ6MWT** | PHQ9 | **100%** | Bootstrap selection from 100 iterations (n=45, α=0.5). Exploratory only. |
| **Δ6MWT** | UT AMP | **99%** | Bootstrap selection from 100 iterations (n=45, α=0.5). Exploratory only. |
| **Δ6MWT** | STEP AFF | **99%** | Bootstrap selection from 100 iterations (n=45, α=0.5). Exploratory only. |
| **Δ6MWT** | ROLL HEALTH | **98%** | Bootstrap selection from 100 iterations (n=45, α=0.5). Exploratory only. |
| **Δ6MWT** | BALO DIM | **98%** | Bootstrap selection from 100 iterations (n=45, α=0.5). Exploratory only. |
| **Δ6MWT** | FRT | **98%** | Bootstrap selection from 100 iterations (n=45, α=0.5). Exploratory only. |
| **Δ6MWT** | 2MWT | **98%** | Bootstrap selection from 100 iterations (n=45, α=0.5). Exploratory only. |
| Green ≥ 80% (stable); amber 50–79% (moderate); grey < 50% (unstable). Exploratory only; not validated in independent sample. | | | |

Statistical notes: Median [Q1–Q3]. Wilcoxon signed-rank (within-group) | Mann–Whitney U (between-group) | Friedman test (repeated measures). Bold terracotta = p < 0.05. — = data not available. Exploratory analyses (S6–S8) not adjusted for multiple comparisons.
